# Experiences of women from ethnic minorities and underserved, marginalised and disadvantaged groups in communicating with health professionals during antenatal care: An overview of qualitative systematic reviews

**DOI:** 10.3310/nihropenres.14096.1

**Published:** 2026-01-07

**Authors:** Kusum Singal, Moira Cruickshank, Aniebiet Ekong, Clare Robertson, Pauline McDonagh Hull, Denitza Williams, Tara Fairley, Louise Locock, Mary Kilonzo, Mo Ade, Lilla Braithwaite, Debra Bick, Declan Devane, Magdalena Rzewuska Diaz, Gillian Taylor, Siladitya Bhattacharya, Mairead Black, Miriam Brazzelli

**Affiliations:** 1 Aberdeen Centre for Evaluation, University of Aberdeen Institute of Applied Health Sciences, Aberdeen, Scotland, UK; 2 University of Aberdeen Aberdeen Centre for Women's Health Research, Aberdeen, Scotland, UK; 3 Cumming School of Medicine, University of Calgary, Calgary, Alberta, Canada; 4 Division of Population Medicine, School of Medicine, Cardiff University, Cardiff, Wales, UK; 5 NHS Grampian, Aberdeen Maternity Hospital, Aberdeen, Scotland, UK; 6 Health Economics Research Unit, University of Aberdeen Institute of Applied Health Sciences, Aberdeen, Scotland, UK; 7 Independent Patient and Public Involvement Partner, Southeast England, UK; 8 Independent Patient and Public Involvement Partner, Lothian, UK; 9 Health Services Research Institute, University of Warwick Warwick Medical School, Coventry, England, UK; 10 School of Nursing and Midwifery, University of Galway, Galway, Ireland; 11 Peterhead Maternity Unit, NHS Grampian, Aberdeen, Scotland, UK; 12 University of Aberdeen School of Medicine Medical Sciences and Nutrition, Aberdeen, Scotland, UK

**Keywords:** Systematic reviews, maternity care, pregnancy, ethnic minorities, disadvantaged women.

## Abstract

**Background:**

Maternal mortality rates show disproportional disparities among disadvantaged groups.

**Objective:**

To conduct an overview of qualitative systematic reviews to summarise the antenatal care experience of ethnic minority and underserved, marginalised and disadvantaged women in high-income countries.

**Search strategy:**

Seven electronic databases were searched to identify reviews published between 2011-2022.

**Selection criteria:**

Two reviewers independently screened search results and full texts of potentially eligible articles.

**Data collection and analysis:**

Data were extracted by two independent reviewers, critically appraised using the JBI tool and assessed for overlap. A thematic analysis was conducted.

**Main results:**

Nineteen qualitative reviews were included. Most were conducted in the UK (n=12) and provided a thematic synthesis of findings. Studied populations included women from minority ethnic groups and those who were migrants, homeless, refugees, asylum seekers, disabled, obese, or had experienced genital mutilation or human trafficking. Common challenges included language and cultural differences, and lack of effective interactions with healthcare professionals. Many women experienced discrimination, isolation, limited awareness of available services and negative attitudes from maternity care staff. Limited access to maternity services was influenced by various factors, including costs and communication barriers. Positive experiences included interactions with culturally responsive healthcare professionals, support from social groups, and access to interpreters.

**Conclusions:**

Our findings highlight the complex challenges some women face during maternity care. Future research should focus on more personalised care solutions, long-term evaluations of maternity services, training of healthcare professionals, and ways to improve the quality of information provided and the interaction with healthcare professionals.

## Plain language summary

This study looked at how women from ethnic minority, underserved, and disadvantaged groups experience communication with health professionals during pregnancy care in high-income countries. These groups include migrants, refugees, asylum seekers, homeless women, women with disabilities, survivors of human trafficking or female genital mutilation, and women with obesity.

Our research team assessed 19 published studies to understand both challenges and positive experiences. Most studies were conducted in the UK. Many women reported difficulties such as language barriers, cultural misunderstandings, and a lack of clear information. Negative attitudes or discrimination from maternity staff made some women feel judged, isolated, or unsafe. Practical issues, such as costs, transportation, and childcare, also limited access to services. In some cases, women felt that the care was rushed, impersonal, or insensitive to their cultural or individual needs.

At the same time, women reported positive experiences when healthcare professionals communicated respectfully, gave clear information, and acknowledged cultural and emotional needs. Access to interpreters, support from family or community groups, and continuity of care also helped improve experiences.

Overall, our findings show that communication is central to good pregnancy care. When women feel listened to, respected, and supported, they are more likely to engage with services and have better experiences. However, many disadvantaged women face significant challenges that increase their risk of poor outcomes.

Maternity services, therefore, need to be more inclusive, culturally sensitive, and personalised. This includes training healthcare professionals in respectful and cross-cultural communication, improving interpreter services, and ensuring better access to supportive networks. Addressing these issues can help reduce inequalities and improve pregnancy care for all women.

## Introduction

Antenatal care plays a key role in the health of pregnant women and their unborn babies ^1^. Effective communication between healthcare professionals and pregnant women during antenatal care is critical for shaping personalised care, addressing specific needs and achieving positive outcomes ^2^. Given the disproportionate adverse pregnancy outcomes in women from underserved and disadvantaged groups, a deep understanding of communication barriers and facilitators is essential for designing inclusive and patient-centred antenatal care services ^3^.

Various studies have shown how underserved and disadvantaged women may experience a range of communication issues during antenatal care, which can result in misunderstandings, mistrust, and dissatisfaction with care, each of which could negatively impact engagement with maternity services ^4, 5^. Healthcare professionals should be aware of the unique needs of underserved and disadvantaged women and take steps to improve their communication and interaction with them, such as providing culturally competent care, using interpreters, and involving family members in decision-making processes. To develop training in these areas, the full range of antenatal care experiences reported by underserved and disadvantaged women in high-income countries should be considered.

This overview of systematic reviews considers the experiences of communication during antenatal care among women from ethnic minority and underserved, marginalised, and disadvantaged groups. Thus, it provides the opportunity to build more culturally sensitive and inclusive antenatal care services. This overview will also inform an ongoing study, which aims to develop a birth plan decision aid for UK antenatal care.

## Methods

This overview of systematic reviews was conducted in line with current methodological standards and the PRISMA 2020 statement ^6, 7^. The research methods were predefined and registered in PROSPERO (registration number CRD42022372831, PROSPERO). This overview forms part of a larger mixed methods programme of research aimed at developing a decision aid for discussing planned modes of birth during routine antenatal care in the UK NHS and comparable healthcare settings (the Plan-A study; Research Registry ID: researchregistry8238). While a previous publication from this programme ( https://doi.org/10.1016/j.xagr.2025.100556.) employed similar methodological approaches and a comparable structure, it addressed a different research focus and reported distinct content and findings.

### PPI involvement

Four Public and Patient Involvement (PPI) partners with lived experience of maternity care, including women from underserved backgrounds, contributed to this study to ensure inclusivity and meaningful engagement. They were members of the PPI panel for the wider Plan-A project, which comprised eight patient partners in total. PPI partners were involved throughout the entire research process, beginning as co-applicants at the grant proposal stage. They attended regular study meetings, contributed to key discussions, and played a central role in shaping the research question and defining the scope of the study. Their contributions were informed by their lived experience and experiential knowledge of maternity care.

Although one partner withdrew in the second year of the project, the remaining three PPI partners continued to make substantial contributions. They were closely involved in interpreting the study findings and reviewed and commented on draft versions of this manuscript. Their input helped ensure that the final outputs were clear, relevant, and accessible to a wide audience.

### Inclusivity

The Plan-A decision aid will support all who become pregnant. See the project's language statement for more information ( https://www.abdn.ac.uk/acwhr/research/plan-a-193.php#panel201).

### Eligibility criteria and search strategies

Table 1 outlines the study eligibility criteria. An Information Specialist developed search strategies using appropriate MeSH and text terms. From January 2011 to December 2022, relevant reviews were searched for in major general and specialised databases (MEDLINE, EMBASE, CINAHL, CENTRAL, MIDIRS, ASSIA, and the Social Sciences Citation Index). Details of the MEDLINE search are available in the data repository (see Data Availability statement); this strategy was adapted for searching other databases.

**Table 1.** Inclusion and exclusion criteria.

	Inclusion criteria	Exclusion criteria
**Population**	Women from ethnic minorities and from underserved, marginalised and disadvantaged groups currently experiencing a clinically uncomplicated pregnancy and women who were previously pregnant and gave birth after 37 weeks gestation. By ethnic minority, underserved, marginalised and disadvantaged groups we refer to women who may experience discrimination or isolation because of personal characteristics including age, sexual orientation, gender reassignment, ethnicity, being pregnant, religion or belief, disability and those who do not have the same opportunities as others in society (e.g., refugees, unemployed, women with obesity).	Population of unselected pregnant women with no specific attention to the experiences of women from ethnic minority, underserved and disadvantaged groups.
**Data of ** **relevance**	Experiences of communication and interaction with healthcare professionals during antenatal care. Experiences of values such as respect, trust and fairness shown by maternity care staff during antenatal care. Data related to any instance of inequality in whether, when and how mode of birth options are discussed with healthcare professionals during antenatal care.	
**Design**	Systematic reviews of primary qualitative studies.	
**Setting**	Studies conducted in settings relevant to the UK (defined as systematic reviews where most studies are conducted in high-income countries based on the classification of the World Bank and with at least one included study conducted in the UK) ^10^.	
**Publication ** **date**	2011 onwards. In the UK, the recommendation from the National Institute for Health and Care Excellence (NICE) that women should have the opportunity to discuss mode of birth options in the antenatal period came out in 2011. Therefore, to inform the Plan-A study, we are interested in antenatal communication reported from 2011 onwards, also ensuring that these reflect recent societal norms (e.g., level of knowledge/access to information).	

### Study selection and data extraction

**Figure 1.**
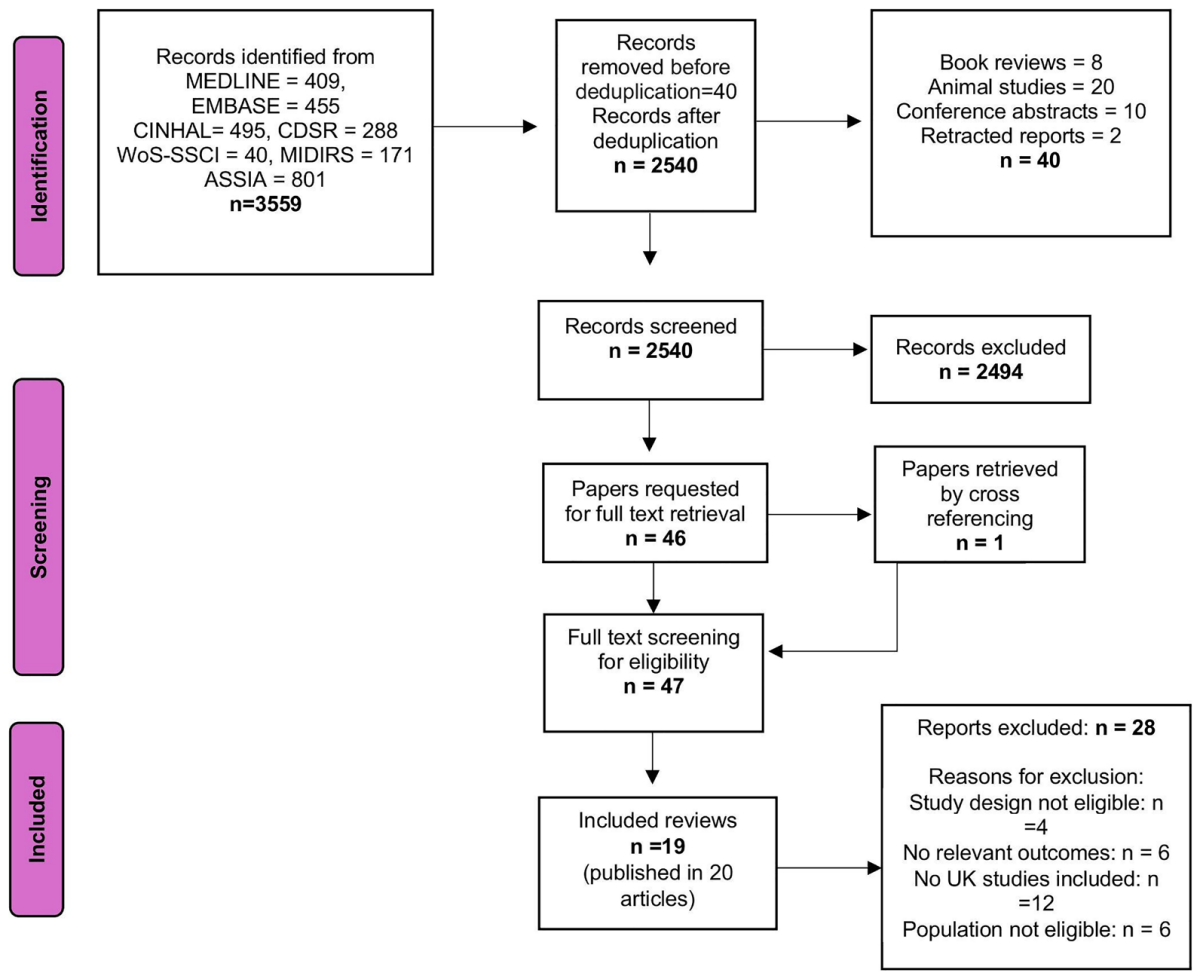
PRISMA Flow chart.

Two review authors (MC and CR) independently screened the search results and assessed the full texts of potentially eligible citations. Figure 1 (PRISMA diagram) summarises the selection process and the main reasons for exclusions. Data extraction was carried out by two independent authors (KS and MB) using a customised Excel form. To ensure consistency, a third author (MC) cross-checked 10% of the extracted data.

### Quality assessment

The methodological quality of the identified reviews was assessed using the Joanna Briggs Institute (JBI) critical appraisal checklist for systematic reviews ^8^. One review author (AE) conducted the assessments, and a second review author (KS) cross-checked them. The original 11 JBI checklist questions were adapted for this overview. No reviews were excluded based on the quality assessment results. Additionally, one review author assessed the overlap of primary studies across reviews using the Graphical Representation of Overlap for Overviews (GROOVE) tool ^9^.

Any disagreements during study selection, data extraction and quality assessment were resolved by discussion between review authors or consultation with the research team.

### Data synthesis

We organised the findings of the included reviews into two main descriptive themes based on our research question: i) barriers and ii) facilitators to positive antenatal care and birth experiences. One review author (KS) conducted a thematic analysis, examining the similarities and differences across the reviews to identify specific barriers and facilitators (subthemes). A second review author (MB) independently reviewed the analysis and subtheme structure to enhance reliability. Both review authors then discussed their interpretations, reviewed participants’ quotes, and considered the authors' conclusions of the included reviews.

## Results

A total of 3,559 citations were identified. After deduplication and removing irrelevant records, 2,540 citations were screened for eligibility. Of these, 2,494 were excluded for not meeting the inclusion criteria, and 46 were retrieved for full-text assessment. Two additional reviews, Balaam 2013 ^11^ and Frank 2021 ^12^, were found by hand-searching the reference lists of the full-text reviews. After assessment, 19 systematic reviews published in 20 papers were deemed suitable for inclusion (see Figure 1).

### Description of the included reviews

All included reviews were published between 2013 and 2022. Two reviews by Higginbottom *et al*., from 2019 and 2020, reported the same data, with the 2020 review selected as the primary source ^13, 14^. Most reviews (11/19) were conducted in the United Kingdom ^11, 14– 22^, four in Australia ^12, 23– 25^ two in Ireland ^26, 27^ one in Canada ^28^, and one in several European countries (Denmark, Finland, Germany, Ireland, Italy, Norway, Portugal, Sweden, Switzerland, The Netherlands, UK) ^29^. Across reviews, the number of primary studies, published between 1990 and 2020, ranged from six to 69. Most reviews used qualitative and thematic synthesis to analyse women’s communication experiences during pregnancy. One review used integrative systematic methods ^28^ and another used a realist synthesis technique ^19^. The methodological quality of primary studies was assessed using various tools: Walsh & Downe’s criteria (n=2) ^11, 16^, SCHEMA (n=1) ^30^, CASP tool (n=9) ^12, 15, 17– 22, 26^, JBI tool (n=2) ^23, 25^, MMAT (n=1) ^28^ and CEBMa (n=1) ^14^. Overall, the primary studies were reported to be of good quality, though three reviews did not provide information on the methodological quality of the included studies ^24, 27, 29^.

The quality of the included reviews, assessed using the JBI appraisal checklist, was generally moderate to high. Two studies were rated as low quality. Most reviews clearly described the research question, used appropriate inclusion criteria, identified relevant evidence, developed effective search strategies and summarised results effectively. In most cases, primary studies were independently appraised by two or more reviewers. However, four reviews exhibited unclear practices or did not follow current standards ^21, 23, 25, 27^. Some reviews also lacked clear recommendations for practice and policy ^15, 18, 22, 27^. Details of the quality assessment are available in the data repository as extended data (see Data Availability statement).

**Figure 2.**
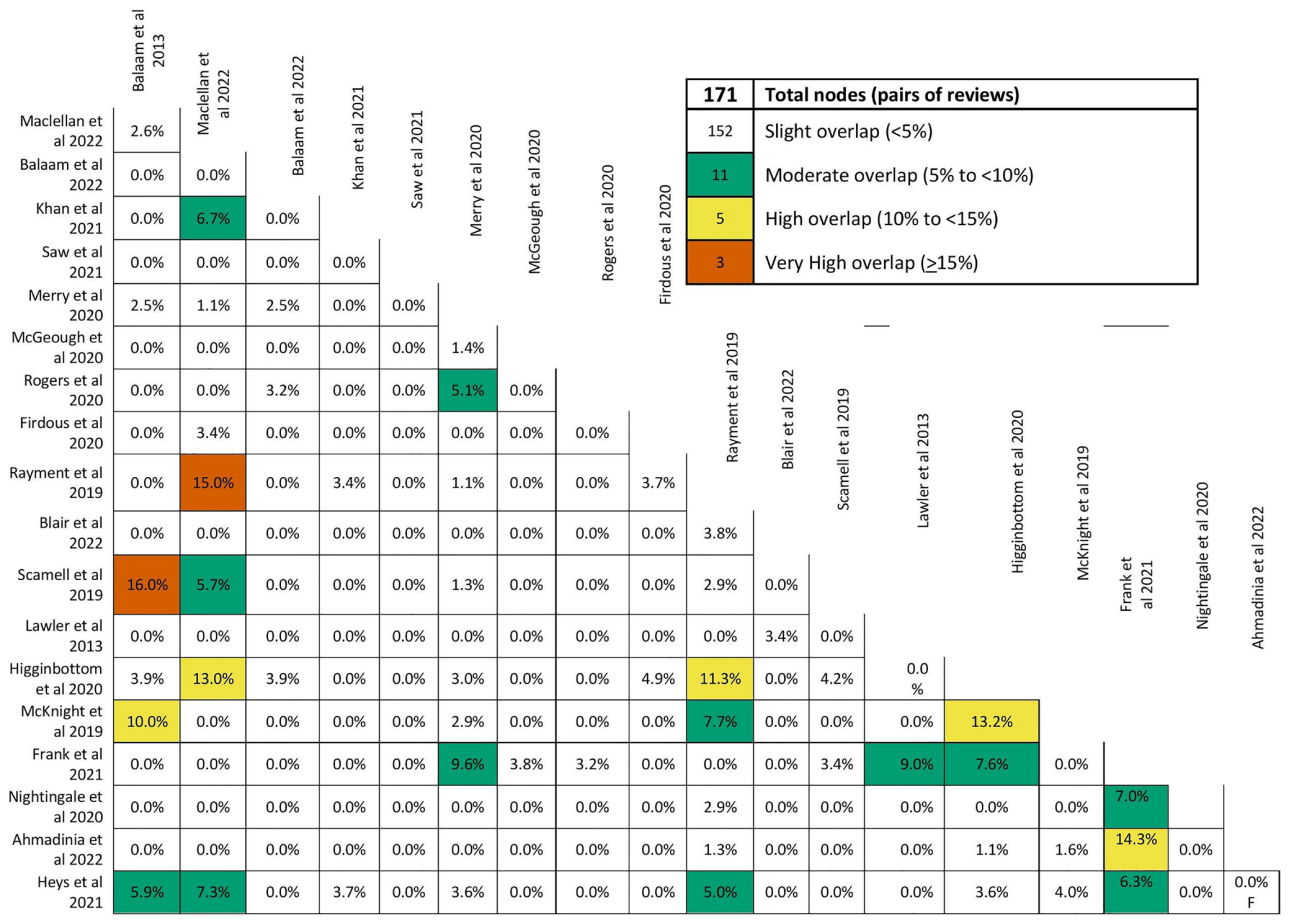
Extent of overlap among systematic reviews.

**Figure 3.**
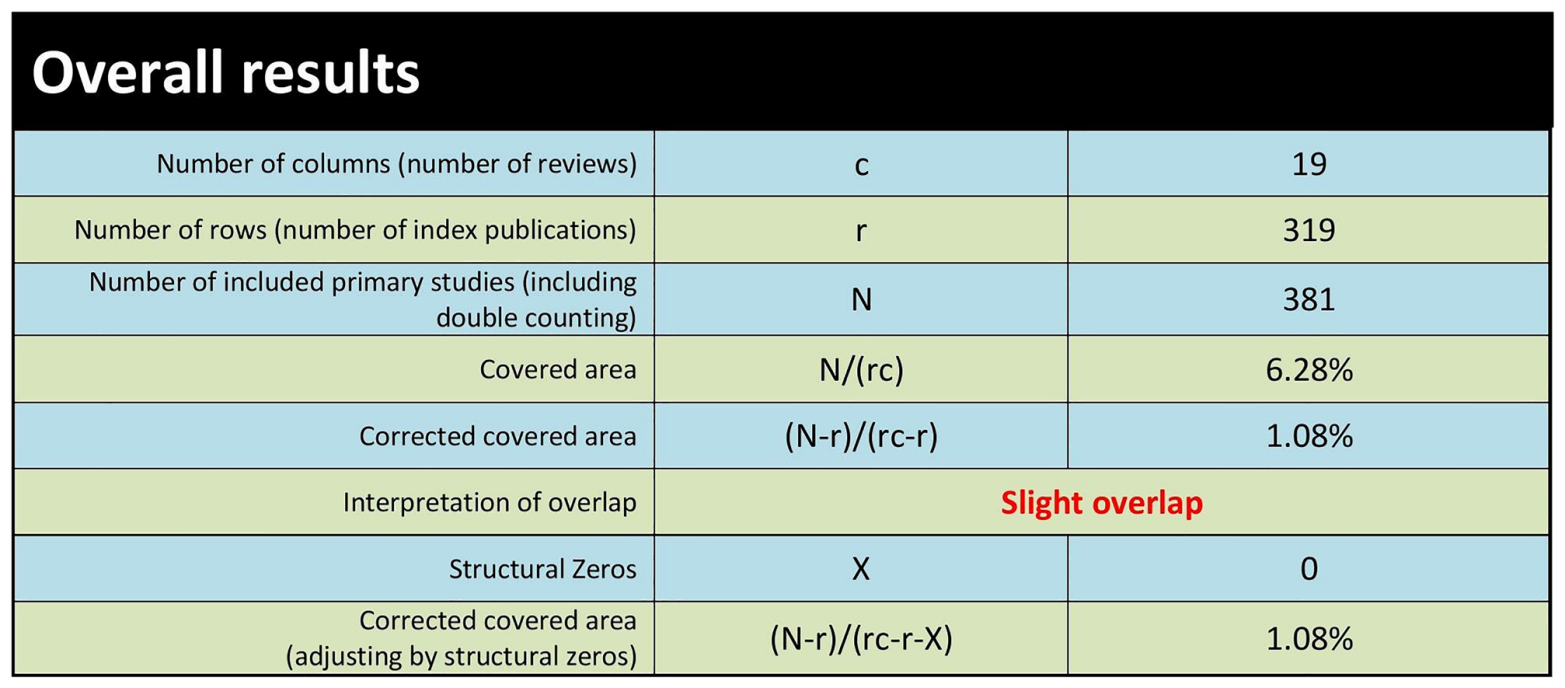
Extent of overlap among systematic reviews: Overall results.

We evaluated the extent of overlap among primary studies included in the identified systematic reviews using the GROOVE tool and found only a minimal overall overlap (1.08%). A total of 381 primary studies were included in the assessment, with a visual representation showing the extent of overlap across reviews. Out of the 171 possible review pairs, 152 shared less than 5% of the primary studies, indicating a minor degree of overlap and a diverse focus. However, 11 pairs had moderate overlap (5% to <10%), 5 pairs had higher overlap (10% to <15%), and three pairs showed very high overlap (sharing 15% or more of primary studies), suggesting some reviews had a more significant degree of similarity or duplication in their content (see Figure 2 and Figure 3).

The 19 systematic reviews included in this overview assessed the experiences of various groups, including immigrant, refugee, asylum-seeking women, and homeless women ^11, 12, 17, 19, 24, 26, 28– 30^, BAME ^21, 22^ and Muslim women ^20^, women with physical disabilities ^23, 27^, disadvantaged women (including those with obesity) ^16, 25^, survivors of female genital mutilation ^18^, and trafficked women ^15^. While all included reviews examined the challenges and barriers faced by women in accessing antenatal care, eight reviews provided also information on facilitators to improve interactions between women and healthcare professionals ^12, 14, 16, 20, 22, 24, 28, 30^. A summary of the major themes and sub-themes identified by the identified systematic reviews is presented in Table 2.

**Table 2.** Summary of findings from the included systematic reviews.

Author & Year	Geographical Location	Population studied	Number of studies and participants	Major identified themes and sub-themes	Quotes related to themes (supportive quotations)	Overall Quality Assessment
Balaam *et al.* 2013 ^11^	UK	Migrant women (refugees, asylum- seekers, illegal, and economic migrants)	495 women from 16 studies (5 from Sweden, 6 from the UK, 2 from Switzerland & 1 each from Norway, Ireland, and Greece)	**Struggling to find meaning in the new country** ➢ Communication and connection Many women described examples of unsuccessful communication with health professionals and the lack of a satisfactory connection with them. Some women reported being afraid to discuss their concerns with health professionals and others reported that they did not receive the information they needed about access to care. ➢ Striving to cope and manage Many women struggled to cope with their new status as migrant pregnant women. They felt insecure, sad, lonely, and isolated. Other commonly reported feelings were vulnerability and anxiety. ➢ Struggling to achieve a safe pregnancy and childbirth Women found it difficult to understand the dominant Western norms and medical philosophy. Concerning giving birth, some women trusted their religious beliefs over medical procedures. Concerns about poor health, HIV, hepatitis, and female genital mutilation as sources of complications for the mother and the baby were often reported. ➢ Maintaining bodily integrity Giving birth in the new country and adapting to new norms, which were very different from their traditional norms, left women feeling powerless, hopeless and with a sense of losing their body integrity. For example, women felt embarrassed and ashamed of being exposed to health professionals during birth especially when their husband was present. **Need for caring relationships** ➢ Sources of strength Feelings of satisfaction and completeness due to giving birth strengthened women’s emotional ties to the baby; women stressed the importance of receiving support from husbands, relatives, and health professionals. ➢ Organizational barriers to maternity care In many cases, the advice provided during antenatal care was experienced as frightening, unhelpful, and lacking adequate knowledge of migrant women's reality (for example about genital mutilation or about their worries, fear and anxiety). Women reported also disrespectful and hostile attitudes from health professionals. In general, women found that maternity services were not customised to the needs of migrant women who often are unable to attend several appointments due to practical and economic reasons. Cultural and language barriers also inhibited access to care. ➢ Nature and quality of caring relationships Women expressed the need for sensitive psychosocial support and counselling and valued the opportunity to have access to trustworthy health professionals who have adequate cultural knowledge and insights into their migration status. Some women felt more confident in dealing with female healthcare professionals.	NR	Good
MacLellan *et al.* 2022 ^22^	UK	BAME women	760 participants from 24 studies	**Giving birth in a technocratic system** ➢ BAME women perceived care to be functional but not supportive. They did not feel respected or being cared for as a person. They felt that the healthcare system was unable to engage with the complexity of their lives. **Poor communication** ➢ Women reported several examples of communication failures due to wrong assumptions and opinions on the part of health professionals. They also felt that health professionals did not have time to explain and signpost key information. In some cases, women highlighted that they had to undergo procedures without fully appreciating their purpose or knowing their risks. In the interaction with health professionals, many women reported cultural and language barriers and interpretation challenges. **Mistreatment of women** ➢ BAME women reported that they were treated in an unsympathetic or unhelpful way compared with the way White women were treated and some women felt discriminated against because of their ethnicity. **Woman-centred care as exceptional, not routine** ➢ Women felt safe and reassured when they had trusting relationships with healthcare staff. Trust was built more easily when the women could rely on continuity of care (the same midwife throughout pregnancy and labour). However, women-centred care was found to be an exception rather than the norm.	*“It was the midwife. She did not* * want to know. She had a set of things she * *wanted me to do, and she did not want* * me to ask any questions. It did not* * matter that I speak English.”* *“…they gave lots of paperwork which, to* * be honest, I do not even think I’ve read* * of it to this day.”* *“I do not know what’s going to happen* * so and I did not know when they do a * *sweep, I did not know what that was.”* *“I was on a ward of four white women, * *and I asked her, ‘You were good with * *the person next door, could you help * *me. I asked two or three times I wanted * *to breastfeed, and they did not come* * to me, yet they helped all the white * *women.”* *“My voice was heard, you know, they took * *my issues to heart.”* *“You know, when you get to know * *someone, it is easier to talk and stuff.”*	Good
Balaam *et al.* 2022 ^30^	UK	Asylum- seeking and refugee women	760 participants from 24 studies	**Alleviation of being alone** ➢ Women’s feelings of sadness, loneliness and isolation were linked to poor communication and language difficulties and to a lack of understanding of how their new society worked. Community initiatives such as social groups and sharing experiences with other women helped reduce the feeling of isolation. **Safety and trust** ➢ Women valued the opportunities to develop relationships of trust with those who supported them. This was either through a one-to-one relationship with health professionals or community helpers/peer supporters or by creating safe spaces in which women could express themselves and feel safe. In general, women felt that peer supporters were more available and less formal than healthcare professionals. **Practical knowledge and learning** ➢ Women valued the opportunity to learn and acquire new knowledge from health professionals and community helpers/supporters during pregnancy. Some women were unfamiliar and not at ease with the cultural practices of the host country in terms of birth and parenting. ➢ Community-based interventions increased women’s awareness of maternity care and led to more women accessing services. Some community-based interventions enhanced women’s experiences by facilitating cultural adaptation. For example, they contributed to changing personal beliefs, gender roles within relationships and also attitudes towards the practice of FGM. **Being cared for and emotional support** ➢ Women reported experiencing depression, anxiety, and fear as they had to face pregnancy in challenging and stressful situations. Continuity of care and emotional support provided a sense of security and of being cared for and were associated with reduced levels of stress and reduced fear of hospital, labour and birth. Women considered opportunities for talking and being listened to as particularly important and felt reassured by spending time with maternity staff, community supporters and doulas. **Increased confidence** ➢ Community-based interventions and the opportunity to talk to women and families facing similar challenges increased women’s confidence in their ability to speak out in some contexts and helped them overcome difficulties related to birth and parenting. ➢ Some interventions also helped women to move forward and take control of their lives with some women becoming supporters for other women.	*“Having someone to talk to at a * *children’s centre where I felt safe made* * all the difference, she saved me.”* *“The midwives don’t have the same* * understanding as us. When we talk with* * the women, it is heart communication.”* *“When I [doula/midwife] arrived and* * spoke the same language as her* * and told her that I would be with* * her throughout the period - during * *childbirth and at the maternity ward * *- she * *felt safe. It was very important for* * her.”* *“I love her so, so, sooooo much! She * *gives me lots of advice, lots of, every* * week when she comes”* *“In Africa, when you have the operation * *[caesarean section], your family and* * friends cry and pray because there is * *a strong chance that you could die, so* * I was terrified and refused to have it,* * but on Baby Steps they managed to* * convince me that it is different here and * *that I would be alright.”* *“I am able to talk about my worries …* * I feel I have known her for 5 years, she * *understands me.”* *“If they weren’t around I would have * *felt scared… they were very helpful and * *comforting”* *“I used to think I was nothing now I * *think I’m something and when I wear* * my refugee council badge I feel like a * *professional.”*	Good
Saw *et al.* 2021 ^25^	Australia	Women with BMI>30 kg.m ^2^	24676 participants from 17 studies	**Inconsistent or absent information regarding weight management** ➢ Many women described that information on obesity-related risks and weight management during pregnancy was lacking, and women's expectations of regular weighing were often unmet. Women also reported a lack of guidance on physical activity requirements, leading to fears about its safety and confusion about weight loss during pregnancy. Misconception about pregnancy as a reason to increase eating was also evident in some women. **Stigma and stereotyping associated with obesity** ➢ In general, women reported being stereotyped by healthcare professionals who inevitably associated overweight with bad lifestyles, eating habits or “laziness”. Women felt uncomfortable during antenatal imaging because of the insensitive comments of the healthcare professionals and were embarrassed to find out that the ultrasound report indicated that the foetus could not be viewed due to obesity. Women reported receiving inaccurate, inconsistent, and negative advice focused on the potential negative outcomes of obesity. **Medicalisation and depersonalisation of maternity care** ➢ Women expressed the desire for a "normal" pregnancy and described how receiving excessive and inappropriate scrutiny and surveillance without clear explanations increased their anxiety. Women also encountered difficulties in fitting into hospital gowns and beds during medical examinations and noticed that the ‘one size fits all’ approach did not work for overweight women. Some women without pregnancy complications complained about over-medicalisation and lack of personalised care. A few women reported feeling coerced into the decision of having a caesarean section. **Desire for information and need for change** ➢ Many women expressed the desire for specific, non-judgmental weight management advice and pointed out that pregnancy should be a time of increased health awareness and motivation for change. Women found that information from leaflets was often too basic, simplistic and not appropriate for pregnancy-specific circumstances. On the contrary, women who attended lifestyle clinics found the advice on weight management useful especially when was tailored to individual needs and personal challenges.	*“I was there flat on my back and the * *ultrasound scanner had pushed and * *crushed my body from the outside and* * the inside to get a view of the baby but* * had to give up. She finally said that it* * was my fault she could not get a good * *view as I was too fat”*. *“I had one doctor who came to see me* * in the hospital. ... I was eating a small* * snack-size bag of cookies, and he walked* * in and just totally scoffed at me that I * *was eating cookies. He said, “It’s things* * like that, I have to tell you . . . I can’t * *even prescribe you birth control because * *you’re too fat for birth control pills.” That * *was real fun for postpartum. I cried for * *about 2 hours after he left.”*	Good
Khan *et al.* 2021 ^21^	UK	Black, Asian and minority ethnic (BAME) women	24645 women from 8 studies	**Communication** ➢ Poor verbal and non-verbal communication and the use of medical terminology represented a major barrier to meaningful interactions between BAME women and healthcare professionals. Women reported feeling dismissed and unsafe because of poor active listening by healthcare professionals. **Midwife-woman relationship** ➢ Women who reported previous negative experiences with midwives found it difficult to build new relationships. The patronising and dismissive attitude of healthcare professionals also generated feelings of isolation and a perceived lack of care. Moreover, the lack of genuine interest in women's emotional well-being from healthcare professionals and busy workloads were a threat to establishing trusted interactions. Women’s limited understanding of the UK health system and midwives’ role resulted in poor quality relationships and engagement. Differences in maternity services and lack of continuity of care affected engagement and late bookings. Ethnic minority women reported experiencing mistreatment and discrimination during childbirth. **Maternity services and systems** ➢ Midwives recognised that short appointment times and limited access to interpreters created barriers for women, leading to delays and difficulties in accessing antenatal care. BAME women showed a lower level of engagement with maternity services, which was often misinterpreted as deliberate avoidance of care. However, reasons that explained women's low level of engagement included transport, domestic, language/ communication issues and differences between the UK maternity services compared to women’s native countries. **Cultural and social needs** ➢ Midwives recognised that some women’s cultural and religious practices did not agree with their health advice and inevitably impacted relationships. Stereotyping and lack of cultural awareness by healthcare professionals contributed to late bookings, skipping of antenatal classes and women’s sense of isolation. Women's requests for cultural adjustments (e.g., to get a female doctor) were not particularly well-received by healthcare professionals. Women’s immigration status was linked to fear of deportation and late bookings or avoidance of appointments. Furthermore, limited social connections and the complex social needs of some women - such as asylum seekers, refugees and trafficked women - affected engagement with maternity services.	NR	Moderate
Merry *et al.* 2020 ^28^	Canada	Migrant families (mothers, asylum seekers and refugees) during pregnancy, postpartum and early childhood.	69 studies	**Communication and Cultural Differences** ➢ Migrant women experienced challenges with communication including poor understanding and culturally inappropriate information. By the same token, healthcare professionals struggled to assess and respond to women’s needs because of language and cultural barriers. Women also had unmet expectations due to the differences in care in their home countries (for example in terms of prenatal visits, ultrasound scans and examinations). ➢ Family separation, including children who remained in the home country, was a source of distress and anxiety. ➢ Some women relied on their own transnational or home country networks to obtain health information and advice. ➢ Initiatives to address cultural, religious and language barriers included the use of community-based doulas/supporters, other mothers from the same community groups and programmes to improve social support and personalised care (including the use of culturally adapted material). ➢ The need for a diverse and trained (e.g. culturally competent) workforce as well as the greater use of linguistic/cultural advisors was also underlined.	NR	Good
McGeough *et * *al.* 2020 ^26^	Ireland	Homeless women	277 women from 7 studies	**Lack of person-centred care** ➢ Accessibility to health system Homeless women were reported to face barriers in accessing antenatal and postnatal healthcare due to a lack of person-centred care and because of the structure of the health system. Barriers included the eligibility criteria to access services and gain insurance status and the fragmented nature of service provision. Fragmentation of service provision was particularly problematic for women who experienced problems with substance abuse, due to the lack of coordination between antenatal health services and other services such as drug treatment. Poor health literacy was also a barrier to accessing maternity care. In general, homeless women showed limited knowledge of reproductive health. Their competing needs made them vulnerable and jeopardised their autonomy. ➢ Attitude of healthcare professionals Attitudes of healthcare professionals were identified as significant barriers to accessing maternity care. Homeless women reported feeling judged by healthcare professionals and treated with less respect and dignity because of their addictions and health issues, or because they did not have health insurance. Feelings of shame, embarrassment, and poor mental health were also commonly reported by homeless women. Negative past experiences, social rejection, lack of support and repudiation by partners and families contributed to creating a sense of disenchantment with life and impacted the women’s willingness to seek care. **Complexity of survival** ➢ For some homeless women distrust in the healthcare system was rooted in their unpleasant experiences in foster care during childhood and in the fear that Child Protection Services could take away their babies. This fear was the reason why most homeless women delayed seeking care. Social agencies were viewed by some women as punitive rather than supportive.	*“I wanted my baby after childbirth, but* * the hospital staff wanted my ID and I* * told her that I don't have it because I lost* * it during my homelessness. They didn't * *even allow me to visit my child.*” *“When providers find out you use drugs, * *they treat you bad, i.e., like an addict.* [They] *put you on the table like you're a* * piece of meat.*” *“I am ashamed because as a woman * *who is useless in this world, why should I* * bring an innocent child into this world?”* *“I was afraid [the clinic] would find out* * about me being pregnant and try to * *take away my baby, just like they do to* * everyone who's been homeless for any* * time during their pregnancy.”*	Good
Rogers *et al.* 2019 ^24^	Australia	Migrant and refugee women	1499 women and 203 service providers from 17 studies.	**Effectiveness of models of care at improving service access** ➢ The role of health advisors or bilingual/bicultural workers was considered important for establishing trusting relationships and providing support and assistance in navigating the health system. ➢ Women who participated in antenatal education programmes provided by bilingual/ bicultural workers showed significant improvements in understanding the importance of antenatal care. ➢ The support of bicultural workers and the cultural safety they provided was perceived as crucial to increasing access to maternity services. **Continuity of care** ➢ Refugee women highlighted continuity of care as a way to reduce the need to revisit their traumatic experiences and valued the opportunity to have more time to discuss relevant issues with health professionals and build positive relationships. **Culturally responsive care** ➢ Culturally sensitive care, psychological support and access to interpreters were considered very highly by refugee women. Some women expressed frustration with "Western" medicalised practices and found discussions about female genital mutilation intrusive and disrespectful. The home visiting programme involving bilingual/bicultural workers was perceived by women as helpful in providing practical and emotional support, case management and education. **Effective communication** ➢ Women appreciated the opportunity to use interpreters and have longer appointments. Health advisors or bilingual/bicultural workers were identified as a means to increase communication between women and healthcare professionals and provide cultural safety. **Flexible and accessible services** ➢ Women considered flexible appointments and the availability of public transport essential for accessing maternity services. ➢ Community-based antenatal clinics alongside social groups and social support services were also considered important to ensure acceptability of care. **Acceptability and appropriateness from the perspective of service providers** ➢ Service providers considered culturally tailored material useful to improve communication with migrant women, especially with Somali women. They also recognised the benefits of continuity of care but found that flexible appointments could increase pressure on health resources. They also emphasised the importance of a holistic social model of health, including acknowledgement of the challenges women face during their migration journey such as isolation from families, emotional and psychological distress, and difficulties in settling in a new country.	NR	Good
Firdous *et al.* 2020 ^20^	UK	Muslim women	142 women from 6 studies	**Islamic practices and individualised care** ➢ In general, there was a lack of understanding and awareness regarding Muslim women’s decision-making process. The presence of men in antenatal classes and within hospital wards impacted women’s engagement with antenatal classes and breastfeeding. Women expressed a preference for Muslim healthcare professionals, noting they would already understand their religious and cultural practices and make them more comfortable to talk about issues related to these practices. ➢ All women also valued continuity of care as a way to build trustable relationships. **Language barriers & lack of awareness about their culture** ➢ Women complained about the lack of awareness of religious values and practices from healthcare professionals and expressed the need for religious-related information. ➢ Women who did not speak English struggled to understand the medical terminology and found that the information provided by leaflets was of little use and did not help their choice. **Injustice, Inequity and Intolerance** ➢ Muslim women felt discriminated against and stereotyped by healthcare professionals who did not understand their religious values and cultural values practices. ➢ Women felt that their clothing such as veil, Hijab [a veil used as a head covering] and Abaiya [a full-length garment] identified them as Muslims and made them prone to discrimination and prejudice. **Spirituality and faith** ➢ Women maintained that their maternity experience was greatly influenced by their faith. Women explained how spirituality helped their mental health and how they used the Quran and called on God when they struggled and needed support. **Positive experiences** ➢ Even though most women reported poor experiences during pregnancy, some women reported positive experiences, especially when they found midwives who understood Islam and when they received personalised care.	*“I don’t like to attend these classes,* * because it is uncomfortable … as it is* * women mixed with men. Where is my* * privacy with these men.”* *“I think with a Muslim midwife you * *would feel more comfortable telling her* * things … you can easily tell aMuslim* * midwife that you want your child to hear * *Allah (God) and she would completely* * understand.”* *“I bought magazines and read about* * birth of baby … When I used to ask my * *midwife for information, she just didn’t* * have time to discuss other things”* *“I could not understand everything they * *said. I told my husband to translate* * everything for me but he did not. He was* * hiding the truth and trying to comfort * *me“.* *“Women who do not speak English..* *that is an issue in our Asian culture you* * know… interpreting is an issue”* *“The manner in which she talked to me* * was very bad … It was clearly because I * *am a Muslim and wear a veil …* *“It is such a spiritual journey …* * motherhood journey would make you* * gain some Iman [faith]” “Without my* * faith … I would probably go through* * depression”* *“I never felt discriminated against on the* * grounds of my race. On the contrary, I* * felt that they respected our religion. In* * the midwife’s first home visit she said* *“Al-salaam alykom” in Arabic instead of* * “hello”.”*	
Rayment- Jones *et al.* 2019 ^19^	UK	Women with social risk factors (asylum seekers, refugees, trafficked women, those experiencing domestic abuse)	22 studies	**Access to maternity services** ➢ Factors that were considered important for asylum seekers, refugees and trafficked women and women who were unfamiliar with the health system, included: • Access to language-appropriate materials and interpreters • Earlier and direct access to maternity services • Ability to access antenatal classes without extensive documentation or fear of being reported to local authorities/agencies. • Ability to rebook missed appointments. **Antenatal education and practical support** ➢ Women expressed a preference for personalised and culturally sensitive antenatal classes and for evidence-based information that was appropriately communicated by health professionals or translated. ➢ They also valued the opportunity to receive practical support. For example, information on how to contact social workers and attend meetings with statutory agencies as well as advice on housing, employment, education and care of children. **Continuity of Care** ➢ Women did not like to have to repeat their stories and difficult circumstances to different healthcare professionals. They appreciated receiving continued support from a known midwife or a small team of midwives. They also valued a more personalised and needs-led approach to care where, for example, appointments are co-planned. **Relationship/trust building** ➢ Women recognised the importance for healthcare professionals to avoid labelling or making assumptions about their needs based on their cultural backgrounds. They valued the opportunity to build relationships with healthcare professionals who were respectful and able to understand their needs and preferences. They also felt it was important to perceive the whole maternity service as safe and respectful. **Overcoming Assumptions** ➢ Women often felt they were receiving paternalistic care and not being able to assume an active role. They felt their cultural needs were easily disregarded in favour of a Western medical model of care. This led to frustration, poor engagement with maternity services and poor uptake of screening and antenatal education. **Surveillance** ➢ Women feared the judgment of healthcare professionals and perceived maternity services as a system of surveillance rather than support, especially those with immigration issues who were worried they could be tracked by authorities or have their babies removed if they registered with maternity or social services.	*“When I was 4–5 months pregnant… I * *snuck out of the house and went to the* * local GP [family doctor] practice. When I* * arrived, they told me* *I needed a passport and proof of * *address. I explained that I didn't have* * this documentation and they turned me * *away”* *“They said to me, until we are sure* * that it's safe you see, to carry on with* * the pregnancy, then you can have a* * booking”* *“I never attended the antenatal class, * *because no one takes care of [my] other* * two kids. Where [can I leave] them?”* *“Not enough information provided they* * give you leaflets and tell you some risks,* * but I would have liked to have talked to* * someone. It is different reading it than* * talking to someone and sometimes you* * don't understand the leaflets. So talking* * is better.”* *“Have one midwife – I think it would be* * much better for me….I can’t be open up* * to a lot …every different people. When it’s* * one person, then you can open up.”* *“I had built a relationship with her. I felt * *looked after and I had confidence in who * *was providing my care.”* *“I thought if you said something how* * you's exactly feeling, and if you was* * feeling a bit down that particular day,* * that they would use that against you.”* *“It is safer not to ask for help, you'd* * better Google rather than ask* * midwives… I didn't want them thinking,* * ‘Oh, she can't do it.”*	Moderate
Blair *et al.* 2022 ^23^	Australia	Women with physical disabilities	27 studies	**Striving for a 'Normal' Pregnancy** ➢ Women with physical disabilities want a “normal pregnancy experience”. They expressed the desire to be cared for in the same way as women without disabilities. Some women reported feeling singled out and did not like to be labelled as ‘high- risk’ because of their disability. However, some women noticed that being treated differently resulted in additional care. **Empowering Independence and Self-Determination** ➢ Women stressed the importance of remaining in control of their decisions even when they experienced exacerbations of their physical disability. They considered it crucial to find healthcare professionals who were respectful of their knowledge and preferences. **Informed Choices** ➢ Women felt more confident in making decisions when they were able to increase their knowledge. Women relied on pregnancy books, research publications and information from disability organisation websites but questioned the validity of information available online. Women reported trusting other women’s knowledge and experience. **Increase Disability Knowledge** ➢ Women complained that healthcare professionals did not provide enough information on the impact that disability could have on their pregnancy. They were also frustrated by the lack of interest and engagement from healthcare professionals. **Healthcare Professionals’ Attitudes** ➢ Many women found healthcare professionals to be insensitive and discriminatory to their disability rights and needs. ➢ Some women reported that healthcare professionals questioned their decision to have a baby and suggested termination or sterilisation. **Maternity care provider communication and shared decision making** ➢ Women with physical disabilities struggled to be in control of their decisions and reported feeling less likely than those without disabilities to feel listened to, spoken to in a way they could understand, and be involved in decisions about their care. ➢ Women appreciated when healthcare professionals respected their right to make informed care choices. **Physical access barriers** ➢ Inaccessible maternity facilities and equipment negatively impacted women’s pregnancy experience. ➢ Inappropriate weighing scales meant that many women were not weighed or not weighed regularly during pregnancy. ➢ The lack of disabled parking spots, ramps, automatic doors, low reception desks, and wide corridors made also it challenging for women to navigate facilities. ➢ The need to improve access to care and services was consistently stressed by women. Some women expressed a preference for midwifery-led care and birthing centres. **Individualised care practices and policies** ➢ Women found maternity facilities guidelines and policies to be inflexible and poorly accommodating. Short and rigid antenatal appointments impacted the ability of women to attend antenatal classes and examinations. When continuity of care was not provided, women found it unnecessary and uncomfortable to repeat their complex medical histories to different healthcare professionals. ➢ Women appreciated healthcare professionals who were considerate of their feelings and needs.	*“I say yes [I was treated differently] in a * *positive way as everything was done to* * make my pregnancy and delivery go as* * smoothly as possible”* *“Find a good doctor that’s willing to work * *with you. If he’s not, you find another”.* *“…have people that have gone through* * it or are going through it and have a* * network. I think having a network of * *peers is most valuable asset that any* * person could have at any point in their * *life. That’s the way for us with disability.* *“Definitely do your research, ask those* * questions, ask questions of the patient.* * If you really want to know about how* * things affect me or certain things, ask* * me as well”.* *“Women with disability have the ability * *and the right to have a child just like * *anyone else, and care providers need to* * not let their own personal views affect* * what advice they give to a patient.”* *“I find being in a wheelchair means I am* * regularly not listened to. My husband or * *mum are asked questions instead of me.* * When the professional does not like what * *I have to say they looked to my mum or* * husband to put me in my place (at least * *that is how it felt).”* *“I was not professionally weighed at * *any time during the pregnancy. Not* * once did they have anyone to weigh me. * *That was another reason why I was like, * *‘You are not putting any drugs into my* * epidural line.’ They were just going to* * approximate my weight”.* *“I had to keep going over the same* * things to different midwives last time.* * This time I have just one midwife and my * *consultant. They know me really well and* * it’s so much better.”*	Moderate
Scamell & Ghumman 2018 ^18^	UK	Women living with female genital mutilation (FGM)	609 women from 12 studies	**Feelings of alienation** ➢ Migrant women, whose FGM had become part of their lives, felt objectified by healthcare professionals and complained about their over-inquisitive and insensitive attitude. Women also reported having their autonomy curtailed by health professionals. Their negative experience with maternity services created a sense of alienation and loss of agency. **Fatalism and divine providence** ➢ Women reported that religious beliefs and faith in God influenced their decisions during pregnancy and childbirth and provided a sense of comfort. ➢ Women from different cultural backgrounds viewed pregnancy and childbirth as natural processes, with some perceiving medical interventions as unnecessary. **Positive and negative feelings about maternity care** ➢ While women appreciated when they received care from attentive and competent midwives, they often felt disrespected and unable to trust their maternity care providers. ➢ Lack of knowledge and skills regarding the management of FGM during birth and cultural differences contributed to generating negative feelings including embarrassment and re-victimization. ➢ Some women also considered the gender of health professionals (male) problematic. **Different understandings of the birth process** ➢ Most women considered childbirth a natural process and struggled to accept that it had to be medically managed. Some women, for example, showed a strong resistance to a cesarean delivery, which was perceived as a potential cause of death. Despite considering childbirth a natural experience, women reported feelings of fear, particularly related to FGM complications. Migrant women's traditional lying-in practices in the postnatal period were not accepted by healthcare professionals, who perceived them as a demonstration of arrogance or laziness. **Feelings about FGM** ➢ Women had contrasting views about FGM. They recognised its harmful effects but also valued its importance as a cultural tradition preserving feminine dignity and social coherence. ➢ Some women reported that their experience of FGM was accompanied by feelings of pride and excitement.	*“All of them just wanted to look at me. * *I didn’t understand why, and nobody * *asked me, but I thought that they found * *exciting to see when I was cut open.”* *"My genitals were on display. A group of * *white-coated staff would come and look * *and talk to each other with disgust."* *“When I go there, I feel like a small girl * *that they are going to take care of. * *They do their work in a good way, but * *you also feel that they want to decide * *everything on your behalf.”* *“The child is a gift from God. If anything * *went wrong during delivery, I would * *never accuse anyone because we know * *that no one wants a bad outcome. If * *something does go wrong, it is God who * *has decided the child’s fate….It is God * *who knows if the pregnancy is going * *well. We do not know. If the baby kicks, * *we are not worried.”* *“I had a lot of questions during my * *pregnancy. I had the feeling there was * *nobody whom I could have really asked. * *I missed a traditional midwife as we * *have in Somalia.”* *“She looked really panicked when she * *tried to deliver the baby, and she didn’t * *know what to do, but I had my niece to * *tell her quickly that she can cut…. I felt * *I was different because of the female * *circumcision I had and wasn’t really sure. * *I felt so embarrassed the whole time* *I was at the hospital.”* *“In my dreams, my delivery and my * *circumcision are sort of mixed up. I * *am lying there pregnant, but only six * *years old, as I was at my circumcision, * *and there are people around me with * *knives cutting me up everywhere. It is * *just awful”*. *“I avoid going to hospital when my * *waters break because of C-section. The * *doctor frightened me by saying you * *may not have a healthy or live baby as * *a result of your FC. I told him I believe in * *Allah who determines my baby’s life… I * *was very scared and afraid.”* *“If you are not married, you have * *problems with your menstruation. If you * *are married and you want to have sex * *with your husband, you suffer from pain.* * If you want to deliver your baby, it is * *difficult. This is terrible!”*	Moderate
Lawler *et al.* 2013 ^27^	Ireland	Women with a physical disability	28 studies	**Challenges to accessibility of services** ➢ Women with a physical disability may not be able to drive independently and have to rely on family members, friends and public transport. Non-flexible appointments are often considered problematic. Other reported challenges include environmental obstacles and unadjustable equipment (e.g., lack of designated parking bays, poor topography, impaired access to toilet facilities, inappropriate height of reception desks and examination tables), and fragmented care (i.e., little collaboration and coordination between services such as physiotherapy and antenatal services). **Challenges preventing high quality of care** ➢ Women with a disability complained about the lack of appropriate and reliable information on which to base their decision. Information was often considered irrelevant, unhelpful and contradictory. ➢ Some women described how health professionals lacked knowledge about disability and pregnancy and were generally uncaring and unable to accommodate diversity. ➢ Women reported also that healthcare professionals had a patronising, domineering and authoritarian attitude towards them. ➢ Women were reluctant to ask questions during antenatal classes as they felt that healthcare professionals and other women could not relate to their anxieties and concerns. **Challenges to acceptability of services** ➢ Women with a disability reported negative prejudicial attitudes toward them. ➢ Often, healthcare professionals wrongly assumed that women with a physical disability were not able to cope with pregnancy and childbirth. ➢ Some women reported receiving the advice to terminate their pregnancy. ➢ Women with a physical disability felt constantly judged by healthcare professionals and were concerned they would be considered a failure in their role as mothers. ➢ Insensitive and derogatory comments from healthcare professionals affected women's self-esteem and sense of autonomy and generated feelings of isolation and exclusion.	NR	Poor
Higginbottom *et al.* 2020 ^14^	UK	Immigrant women	40 studies	**Access and utilisation of maternity services** ➢ Late antenatal bookings and low attendance rates were often observed among immigrant women. Difficulties in accessing antenatal services included low levels of English, immigration status, frequent relocations, and lack of understanding of how maternity services operate. ➢ Other reported reasons included family size, financial situation, geographical proximity to the services, ability to use public transport, legal requirements and preoccupation with their asylum-seeking process. ➢ Women with female genital mutilation (FGM) were more likely to experience problems in accessing services. ➢ Some women felt that childbirth was unnecessarily medicalised in the host country while they considered it a natural process. ➢ Some women avoided going to antenatal classes because they were offered only in English and because they found male presence inappropriate. ➢ Other women were concerned that their husbands, who were used as translators, were not able to communicate their issues properly. **Maternity care experiences** ➢ Women reported good experiences of maternity care when they found caring, kind and helpful health professionals. On the contrary, poor and negative care experiences were linked to racism, indifference, rudeness, disrespect, culturally insensitive attitudes and ineffective communication on the part of healthcare professionals. ➢ Some women, especially asylum-seeking women, perceived some healthcare practices as coercive. ➢ Because of negative experiences with healthcare professionals, women tended to avoid accessing maternity services. ➢ Some women reported more positive birth experiences in their native countries than in the UK. **Communication Challenges** ➢ Limited English-language proficiency and the use of complex medical terminology created communication challenges in the interaction with healthcare professionals. ➢ Misunderstandings in non-verbal communication (e.g., facial expressions and gestures) due to cultural differences were also reported. ➢ Limited awareness of available services and miscommunication resulted in poor access to maternity care. ➢ Women felt disempowered and isolated with regard to decision-making because of the challenges in understanding clinical procedures and outcomes, and the poor communication with health professionals. **Organisation and legal entitlements impacting maternity care experiences** ➢ In general, women reported mixed experiences of maternity care. Positive experiences included feeling safe in giving birth at a hospital facility and not at home, being able to reach the hospital on time because of its proximity, and being able to access good postnatal care. Negative experiences included a lack of continuity of care and limited awareness of the way maternity services worked. ➢ Some women found maternity services too bureaucratic and with a propensity to medicalise childbirth, which was viewed as a natural process. ➢ Women's access to maternity care was influenced by their legal status. Women without entitlement to free maternity care were unlikely to access antenatal care due to confidentiality issues related to their legal status and inability to pay service charges.	*“The Home Office put me in detention * *centre so I could not attend my * *appointments. There were no maternity * *services there for me for the 2 months I * *was there. I was offered appointments,* * but they were cancelled at short notice * *without anyone telling me why.”* *“I just felt that my husband could have * *been better prepared if he knew, you * *know, what area to support me in.”* *“When they wanted me to stay in * *hospital because of my high blood * *pressure I refused . . . I went home . . . * *They took me by force. They rang the * *police and told them to bring me back to the hospital.”* *“I was referred to another hospital,* * they did not advise why. At my third * *appointment, I had an interpreter. * *The whole process was rushed, I did * *not know what to expect. I did not * *feel involved in any of the decisions * *– someone else always made them for * *me.”* *“They [health professionals] should not * *make the assumption that they [Muslim * *men] are going to be present at the * *birth. My husband was not there, I didn’t * *want him there. My mother was there.”* *“I feel I am treated like the air.”*	Good
McKnight *et al.* 2019 ^17^	UK	Asylum- seeking women	89 asylum seekers and 1 refugee from 6 studies	**Communication challenges** ➢ Language barriers were a major obstacle to communication between women and healthcare professionals. ➢ Poor communication was found to be a crucial issue during labour where women struggled to understand what was happening to them. ➢ The need for written information about care and entitlements during pregnancy was also emphasised. **Isolation** ➢ Feelings of social and financial isolation were often reported by asylum-seeking women. ➢ Women reported experiencing emotional difficulties during labour because of the separation from families and friends and the lack of social networks. **Mental health challenges** ➢ Women’s reported poor mental health due to previous trauma and oppression including rape, domestic violence, torture and human trafficking. **Professional attitudes** ➢ In general, women regarded relationships with midwives as positive, particularly in community and specialist services settings. ➢ Some women complained about the lack of awareness of healthcare professionals about their situations and their wrong assumptions (e.g., the assumption that the women wanted to terminate their pregnancy because of their asylum-seeking status). ➢ Some women reported they were treated differently from the home population because of their asylum status. **Access to healthcare** ➢ Language barriers and communication challenges impacted women’s ability to access maternity care. ➢ Women’s lack of understanding of maternity services and the role of healthcare professionals were also reported as barriers to accessing care. **Effects of dispersal** ➢ UK Home Office's mandatory dispersal policy was reported to disrupt women’s maternity care leading to potential health risks, treatment delays, and unnecessary repeated medical screenings. ➢ The policy often resulted in women being moved against medical advice. ➢ The policy of dispersal had detrimental effects on women’s mental health and caused stress, anxiety, feelings of powerlessness, and disruption of social networks. **Housing challenges** ➢ Women felt that the provided accommodations were poor and not safe. ➢ Some women described their housing conditions as cramped, dirty, unhygienic and unsuitable for antenatal and postnatal experiences. ➢ Women also reported that access to personal hygiene products was sometimes denied and the food provided was often inedible, culturally inappropriate or unsuitable for pregnancy. ➢ Strict mealtime schedules resulted in women missing their meals due to lengthy or inappropriate antenatal appointments.	NR	Good
Nightingale *et al.* 2020 ^15^	UK	Trafficked women	13 studies	**Access** ➢ Trafficked women were prevented from accessing health care because of the controlled situation they were in and the abuses they experienced. ➢ Healthcare staff were not always aware of the entitlements to health care that trafficking victims had. ➢ Trafficked women were erroneously refused access to health care due to the lack of identification documents. ➢ Interpreters were often not available and independent professional interpreters who could be trusted by victims were rarely used. **Person-centred** ➢ Some trafficked women reported feeling that they were often perceived as sex workers rather than victims of trafficking. In certain cases, healthcare professionals were described as abusing their professional roles by collaborating with traffickers to provide healthcare services to these women, ensuring they remained hidden and undetected. ➢ Confidentiality was appreciated by trafficked women who did not want to talk about their past experiences, which they found painful and distressing. ➢ Continuity of care provided by healthcare professionals was highly valued by trafficked women. ➢ Trafficked women appreciated healthcare professionals’ kindness and support, including their attempt to speak their language and their initiative to provide clothes and equipment for the babies. **Poor health** ➢ Trafficked women were reported to face numerous health issues and health inequalities. ➢ Sexually transmitted diseases, repeated miscarriages and mental health problems were common among women experiencing sexual exploitation. ➢ Women were reluctant to seek care because they feared the reactions of their traffickers or were scared to be reported to authorities, such as police and immigration services. ➢ Women also feared reprisals for themselves or their families if they revealed their experiences and the circumstances in which they were living.	*“He told staff that I can’t speak any * *English … he will interpret for me and he * *told them some story … the doctor asked * *me directly as well … I didn’t want to say * *it was this person because he was there * *with me”* *“I had no interpreter and so I couldn’t * *understand what happen to me, what * *happen to my health.”* *“I want to forget what happened. I just * *want to move on. I just want to get my * *own flat and live and maybe get a job.”* *“Once a month she [health practitioner] * *sees me. She will sit for at least half an * *hour talking to me. She encourages me.”* *“I miscarried two times in 5 years and * *had to be hospitalised both times due * *to heavy bleeding. All this was very * *expensive and the gharwalli [brothel * *keeper] charged money for all this.”*	Moderate
Ahmadinia *et al.* 2022 ^29^	Europan countries (Denmark, Finland, Germany, Ireland, Italy, Norway, Portugal, Sweden Switzerland, The Netherlands, UK)	Immigrants, asylum seekers, and refugees (including pregnant women)	57 studies	**Access to health services** ➢ Immigrants, asylum seekers, and refugees explained that their choice and use of healthcare services was influenced by factors such as migration status, length of residency, cultural norms and values. ➢ Asylum-seeking women also reported a major impact of behavioural factors and language barriers on accessing maternity services and information. ➢ They also revealed a struggle to understand the structure and function of the national health system and navigate its services. ➢ Immigrants stressed the importance of getting access to fact-based, easy-to-read health information. ➢ Some immigrants reported seeking advice and contacting doctors in their home countries due to the negative attitudes of healthcare professionals, lack of knowledge of the health system, lack of social networks, poor language skills, and fear of being deported by the police. **Communication challenges** ➢ Communication problems between women and healthcare professionals. were grounded in linguistic difficulties and cultural and religious differences. ➢ Some asylum-seeking women felt that they did not receive sufficient information and complained about poor communication with healthcare professionals, inadequate information and lack of clarity regarding procedures and examinations. ➢ Trust was reported to be a key factor in facilitating communication between women and healthcare professionals. ➢ Social networks and transnational health networks were also reported to play a key role as communication media.	NR	Moderate
Heys *et al.* 2021 ^16^	UK	Disadvantaged and vulnerable women	593 women from 20 studies	**Prejudiced and deindividualized care** ➢ Women reported experiencing judgmental, unpleasant and disrespectful attitudes from healthcare professionals about their choices and preferences, their religious values and practices (e.g. wearing a hijab in labour), their social status and personal history and their sexual orientation. ➢ Some women reported feeling embarrassed and humiliated when healthcare professionals made inappropriate and prejudicial comments and when they did not acknowledge their sexuality and personal relationship (e.g. being a lesbian). ➢ Disadvantaged women described how thoughtless and inappropriate comments of healthcare professionals impacted their self-esteem and confidence negatively and left them feeling disempowered. **Lack of cultural sensitivity** ➢ Women were afraid to be mistreated because of their cultural and social backgrounds and some women felt that their needs were not taken into account. ➢ Some women expressed a preference for a female doctor. **Poor emotional connections** ➢ Women described poor emotional connections with their healthcare professionals with some women reporting feeling ‘processed’ or ‘punished’ rather than supported. ➢ Some women also reflected on how the poor relationship with healthcare professionals impacted negatively on their views of maternity care and made them feeling disengaged from the birth process. **Demoralising interactions & neglectful care** ➢ Some women complained about paternalistic and demoralising interactions with healthcare professionals and the lack of attentive and respectful care. ➢ Some women described examinations that crossed into neglectful or abusive care (e.g. when healthcare professionals refused to stop painful procedures that resulted in traumatic experiences for women). ➢ Negligent care was also reported by women who survived human trafficking. ➢ Some women described their negative experiences accessing maternity care and reported feeling pressured into making decisions. ➢ Some women reported feeling they had no choice or say in relation to examinations and procedures. Other women described how healthcare professionals used the threat of danger to ensure women conformed to their decisions. ➢ Some disadvantaged women also felt that they were not treated as other women and felt discriminated against. **Being heard** ➢ Effective communication, continuity of care, and acknowledgement of individual preferences and needs were among the key factors women reported to describe a positive maternity care experience. ➢ Non-verbal interactions were also valued by women, especially those where English was not their first language.	*“I told [my midwife] I didn’t like going * *to my appointments, and one day she * *just asked me, ‘do you do crack?’… * *Just because I don’t want to come to * *my appointments, I got to be a drug * *addict?”* *“I’ve had a lot of issues in the past * *with people telling me I’m not good * *enough….but that’s exactly what they * *were doing, making you feel like you was * *not good enough.”* *“If the nursing staff see you are foreign * *or of a different colour, they treat you * *badly.”* *“There was a male who entered my * *room, I also put a sign on the door, but * *they didn’t respect it. This man came and * *saw me. I was very upset and crying.”* *“Every time I saw the midwife during * *pregnancy and labour, I felt that I was * *just being processed, there was no * *opportunity to develop a relationship.”* *“I understand there is a staff shortage * *and staff are under a lot of pressure but * *attitudes should remain sympathetic * *towards mothers.... as giving birth can * *be very traumatic and care received has * *a lasting effect on their lives and views * *about hospital care.”* *‘‘Get your life together’. I thought to * *myself, She’s very unprofessional. My life * *is together.”* *“An internal examination at nine months * *was so rough it made me bleed, and * *worse, was so painful and frightening I * *felt I had been assaulted”* *“She [the midwife] did not explain that to * *me. She just started to put - and when I * *shouted, she - she didn’t explain nothing * *to me. Oh my God.”* *“When I saw her with the other women * *in the hospital and she was so respectful: * *‘What do you want to do’, and ‘It’s your * *baby?’ Not like with me.”* *“The best thing the midwife did for me * *was to sit by the bed, at eye-level, hold * *my hand, and acknowledge me. That * *was the best in order for me to feel * *secure as a woman - that I was heard.”*	Moderate
Frank *et al.* 2021 ^12^	Australia	Asylum seekers	116 women seeking asylum from 8 studies	**Communication barriers** ➢ Asylum-seeking women reported facing significant communication problems in their interaction with healthcare professionals during antenatal care. They explained that because of language differences, they were never sure they understood what healthcare professionals were trying to say. They complained about the lack of professional interpreters and the dismissive and disrespectful attitudes of healthcare professionals towards their needs. **Feeling ignored and isolated** ➢ Asylum-seeking women described missing the support of family and friends and reported feeling isolated and alone during the antenatal period and childbirth. ➢ Some women reported being ignored because of their status as asylum seekers and found it challenging to access maternity care in a timely manner. ➢ Because of negative maternity care experiences and fear of deportation, some women made the decision to delay care or avoid antenatal and postnatal appointments. **Dislocation and relocation** ➢ Because of dispersal policies in some countries, asylum-seeking women explained they had no opportunity to understand the health system and build trusting relationships with healthcare professionals. They described the stress and sense of insecurity and uncertainty generated by recurrent relocation. Some women reported having to repeat tests and examinations every time they were relocated. **Positive maternity care experience** ➢ Asylum-seeking women described positive maternity care experiences when they felt supported by empathetic and caring healthcare professionals. Some women reported feeling heard, empowered and acknowledged by compassionate healthcare professionals.	“ *I asked them, ‘[can] we cancel the * *meeting until we get an interpreter. I * *didn’t understand you, and you didn’t * *understand me.’ She [the midwife] * *said, ‘No, it’s okay, we can go on, you * *understand English.”* *“They [midwives] communicated in sign * *language, and I was never sure if I had * *understood properly.”* *“I was worried that something was * *wrong with the baby who was just * *screaming and screaming. After a long * *while, staff entered and said something * *incomprehensible…and then just left * *again. I was hoping that she was going * *to come back again with an interpreter. * *That never happened.”* *“Just crying, just thinking, I have just * *me, why [is] my mum not here, or * *my cousins, or my friends. My sister. * *Nothing.”* *“Sought care for severe pains, I had * *waited from twelve in the day to * *twelve at night. We did not receive any * *examination. We felt ignored and drove * *home.”* *“It would be better if I could have stayed * *in one place. Moving around made me * *feel sad, tired, and unhappy.”* *“I had to start again from zero, I was * *pregnant and I was sicking [vomiting] * *all the time. They bring me here. I didn’t * *have nobody here.”* *“The best thing the midwife did for me * *was to sit by my bed, at eye-level, hold * *my hand and acknowledge me. That was * *the best in order for me to feel secure as * *a woman – that I was heard.”* *“When I saw V [community midwife] * *[had] come [to] see me, I was like all my * *family [had] come to see me.”* *“I know there’s someone who’s listening * *and understanding, which makes me * *feel better.”*	Good

### Theme 1 – Barriers to positive maternity care and birth experience

Within this overarching primary theme, four subthemes were identified. These highlight the challenges faced by ethnic minority and underserved groups during antenatal care, emphasising the need for person-centred, culturally sensitive, and equitable care. Some barriers, like language barriers, are common across subthemes.

**1**. **Communication challenges**
  Across reviews, women described various communication issues with healthcare professionals. These include language and cultural barriers leading to misunderstandings that threatened the quality and value of the interaction with maternity care staff and affected their experiences of antenatal care. Language barriers, in terms of English competence and fluency, were particularly problematic for ethnic and marginalised groups, with 14 of 19 reviews highlighting this issue ^11, 12, 14– 17, 19– 22, 24, 28– 30^. The lack of interpreters and the use of complex medical terms led to misunderstandings. Women faced significant challenges in navigating the maternity care system and understanding the available services. Many reported feeling alone, isolated, and hesitant to ask questions or disclose their symptoms. They often expressed uncertainty about how the healthcare system operated, further complicating their ability to access appropriate care.
**2**. **Attitudes of healthcare professionals**
  Women from ethnic minorities and underserved, marginalised and disadvantaged groups often faced discrimination, disrespect and negative attitudes from healthcare professionals linked to personal characteristics such as ethnicity, disability, and immigration status. Fourteen reviews noted that women’s experiences, including lack of emotional support and culturally insensitive care, negatively impacted women’s maternity care experiences, leading to feelings of loneliness, anxiety, and diminished self-esteem ^11, 12, 14– 18, 20– 23, 26, 27, 29^. Previous negative experiences with the healthcare or social care system hindered women's ability to form meaningful relationships, leading to feelings of isolation and fear ^21, 26^. Some women reported judgmental and derogatory comments from healthcare professionals about their age, sexual orientation, ethnicity, physical characteristics, social status, and birth preferences ^12, 14, 16, 20, 22, 23, 26^. These prejudicial remarks lowered their self-esteem and made them doubt their ability to be effective mothers ^27^. Some reviews highlighted a lack of "respectful" care, with some women's experiences crossing into abusive and negligent care ^14, 21^. Women reported that healthcare professionals were sometimes insensitive to their cultural, religious, and social needs, failing to understand the differences between their native maternity care system and that in the UK ^13, 21^. For instance, Muslim women reported finding the presence of men in antenatal classes uncomfortable ^20^. These cultural mismatches led to clashes with healthcare professionals and influenced women’s decisions to use maternity services. The lack of culturally aware care made many women feel mistreated or fearful of mistreatment ^16^.
**3**. **Access to and experiences of maternity services**
  Fifteen reviews discussed the challenges that women from ethnic minorities and marginalised groups face in accessing antenatal care, such as immigration status, language and cultural barriers, and difficulties in navigating the healthcare system ^11, 12, 14– 17, 19, 21, 23– 27, 29, 30^. Migrant and refugee women struggled to access and maintain continuity of maternity care due to factors such as loss of social status and family support, low self-esteem, and insecurity about their identity ^11, 12, 19, 24, 30^. Economic and practical challenges were also reported, including travel costs, ineligibility for services, childcare needs and environmental barriers ^23, 27^. Reviews noted that local services often failed to meet the needs of those women who were late in registering for appointments and struggled to keep regular attendance due to language and cultural barriers, economic and social circumstances, limited social connections, and immigration status complications ^11, 14, 17, 19– 21, 26, 29^. Issues such as rushed appointments, inadequate provision of information, fragmented care, medicalisation and lack of personalised care were reported to be distressing and worrying ^11, 14, 21, 26^. Medicalisation, excessive scrutiny and dietary counselling were also reported to be upsetting by obese and immigrant women ^25^. The maternity system was frequently described as functional but not supportive, with healthcare professionals treating women as mere cases rather than individuals. As a result, women felt more 'processed' than genuinely cared for ^16^. Short staffing and high workloads further impacted care quality.
4. **Trust and sense of security**
  Twelve reviews examined how safety and trust issues and legal concerns affected women's sense of security in the healthcare system ^12, 14– 20, 26, 27, 29, 30^. Some women explained they were reluctant to seek care or share personal information due to fears for their safety or that of their families. Distrust in healthcare professionals often stemmed from negative foster care experiences or the fear that Child Protection Services would take their babies away ^26^. Homeless women in particular, delayed seeking care due to viewing social agencies as punitive. Fear of childbirth, tension over traditional postnatal practices, and concerns about legal status, also influenced women’s attitudes toward maternity care ^14^. Immigrant women, including homeless, asylum seekers, and refugees, struggled with maintaining self-identity and bodily integrity and felt uncertain about what to expect during pregnancy, birth and after birth.

### Themes identified for specific ethnic, underserved, or marginalised groups

In addition to the above-reported themes, we identified subthemes specific to certain ethnic, underserved, or marginalised groups. Two systematic reviews focused specifically on women with disabilities ^23, 27^, one on women with obesity ^25^, one on women with female genital mutilation ^18^, one on trafficked women ^15^, and one on Muslim women ^20^. These subthemes are summarised below.

### Women with disability

Desire for a normal pregnancy experience
  Women with physical disabilities expressed the desire to be treated as any other pregnant woman and not be labelled ‘high-risk’ because of their disability. They also stressed the importance of remaining in control of care decisions irrespective of the fact that during pregnancy, they could experience exacerbation of their physical symptoms ^27^.
Accessibility Barriers
  Women with physical disabilities reported negative maternity experiences because maternity facilities were inaccessible to them or not properly equipped, such as the lack of adjustable tables and accessible weighting scales ^23, 27^. Many felt these barriers limited their independence and caused anxiety ^27^. They also expressed frustration over being unable to attend antenatal classes due to how they were organised, such as the need to move on and off the ground ^23^.

### Women with obesity

Lack of accurate information on weight management
  Many women with obesity explained they did not receive adequate advice on weight management, obesity-related risks, or physical activity requirements during pregnancy. Some said the topic was often ignored by healthcare professionals, leaving them confused and unsure about whether losing weight during pregnancy was healthy or how it could be achieved ^25^.
Stigma and stereotyping
  Women with obesity often described their interactions with healthcare professionals as embarrassing and humiliating due to obesity-related stigma. They questioned the accuracy of some medical advice and felt uncomfortable as conversations shifted from their pregnancy to their weight. Health professionals’ negative comments about obesity-related pregnancy risks left many women feeling guilty and devastated. During ultrasounds, they were embarrassed when obesity prevented visualisation of the foetus, especially when this issue was never mentioned during previous consultations. Extra tests and referrals caused anxiety and the sense that their pregnancy was not "normal." Some women even avoided antenatal appointments due to the insensitive manners and even bullying attitudes of healthcare professionals.

### Women who underwent female genital mutilation

Sense of alienation and being objectified
  Women who had undergone genital mutilation often reported feeling alienated when seeking maternity care. For migrant women, this feeling of alienation was worsened by healthcare professionals' lack of cultural sensitivity and understanding. Women described being shocked by the intrusive questioning about their genital condition and often felt objectified or subject to disrespectful examinations, with little control during antenatal consultations. Clinical decisions often overrode their preferences, leaving them feeling stripped of autonomy ^18^.

### Trafficked women

Access to care and safety issues
  A major barrier for trafficked women in accessing healthcare was the fear of being reported, arrested, or deported. Understanding and navigating the healthcare system could also be challenging due to being controlled by traffickers and lacking freedom of movement. As a result, they often sought maternity care late or only in emergencies. Some were forced to undergo illegal pregnancy terminations, facing an increased risk of complications and death. Healthcare professionals were not always familiar with the legal rights of trafficked women and tended to deny care if identification was incomplete ^15^.

### Muslim women

Spirituality and faith
  Muslim women explained that their pregnancy choices, such as declining certain screenings, were influenced by their Islamic faith, which played a prominent role in their lives. However, they felt that healthcare professionals disregarded or disrespected these religious beliefs ^20^.
Discriminatory behaviour and lack of cultural awareness
  Some Muslim women reported poor maternity care due to discriminatory and negative attitudes of healthcare professionals, particularly midwives. They felt their clothing, like veils and Hijabs, made them targets for prejudice. Many felt uncomfortable discussing birth plans, as midwives lacked an understanding of Islamic values and practices ^20^.

### Theme 2 - Facilitators to positive maternity care and birth experience

Eight of the included systematic reviews identified factors to enhance effective communication and antenatal care for women from ethnic minority, underserved and marginalised groups ^12, 14, 16, 20, 22, 24, 28, 30^.

**Interaction with healthcare professionals**
  Women generally reported positive antenatal and birth experiences when healthcare professionals met their emotional and psychological needs and adopted caring, responsive, and respectful attitudes ^14, 30^. Positive experiences included clear and respectful communication, receiving appropriate birth information, and continuity of care ^13, 16, 20, 22, 24^. Women, particularly from ethnic minority groups, felt reassured when they had access to interpreters, culturally responsive professionals, and information in their native language ^24, 28^. Some, such as Muslim women, reported feeling more comfortable with healthcare professionals who shared their cultural background ^20^.
**Social and emotional support**
  Asylum seekers, refugees, and migrant women found educational, community-based, social, and peer support groups useful to tackle challenges like social isolation, poor mental health, and housing, financial, and legal issues ^14, 30^. These groups provided a safe space for building trusting relationships and sharing knowledge and experiences ^22^. The involvement of mentors, health advisors, bilingual/bicultural staff, and female staff was key in building trust, offering social and emotional support, and improving access to antenatal information and care ^22, 24^. Family members, especially partners, were also seen as a positive influence, encouraging women to seek maternity care ^26^.
**Access to maternity services**
  Key factors reported to improve access to and experience of antenatal care included the location of services, availability and cost of transport, appointment scheduling (time, length, and flexibility), and the provision of a social model of care ^24^.

## Discussion

This is the most comprehensive and up-to-date summary of evidence from qualitative systematic reviews on the barriers and facilitators experienced by women from ethnic minorities, underserved, and marginalised groups during antenatal care. We identified 19 qualitative systematic reviews on women's maternity care experiences, with the overall methodological quality rated as moderate to high.

Language barriers, cultural differences, and unfamiliarity with the healthcare system significantly impacted how women accessed and engaged with maternity care. Migrant, refugee, and ethnic minority women, in particular, faced challenges in establishing effective communication and trusting relationships with healthcare professionals, which became a major concern during pregnancy. Currently, interpretation services and multilingual information are often insufficient or unavailable in many UK centres. Policymakers should prioritise strategies to increase interpreter use and provide culturally appropriate social support, which is vital for migrant and ethnic minority women. These women face high levels of stress and vulnerability, struggling to adapt to the country's social norms and services. This overview highlights that many women miss antenatal appointments due to difficulties in navigating the healthcare system. Policymakers should improve information on maternity services and create tailored communication strategies.

The attitudes of healthcare professionals towards women from ethnic minorities, underserved, and marginalised groups were considered a major barrier to accessing antenatal care. Women felt they were treated with less dignity, respect, and attention compared to others, due to their cultural or religious background, deprived social status, ethnicity, or physical appearance. Some women, such as those with FGM or obesity, described feeling shocked and humiliated by rude, insensitive, and intrusive comments from healthcare professionals about their physical characteristics.

There is a clear need to improve the understanding and attitudes of maternity staff and policymakers toward women from ethnic minorities, as well as underserved, marginalised, and disadvantaged groups, including migrants and refugees. Future research should explore organisational models that consider these diverse needs and build partnerships with immigrant communities ^31^. Adopting an inclusive, individualised care approach, including cross-cultural training for healthcare professionals, could improve engagement with vulnerable women and better address their complex needs. Training of health professionals is essential, as these women often face severe mental health issues due to personal stressful circumstances and uncertainty about their future ^32^.

Effective communication, meaningful interaction with health professionals, and consistent continuity of care were key factors linked to a positive antenatal care experience. For women with past traumatic experiences or negative interactions with healthcare professionals, consistent, supportive care can help build a trusting and safe relationship during pregnancy, especially when prior experiences have diminished their confidence in the healthcare system. In 2021, NHS England introduced guidance for Local Maternity Systems and Integrated Care Systems to implement continuity of care as a standard model for all pregnant women ^33^. The midwifery continuity of carer (MCoC) model prioritises those at higher risk of poor outcomes. With the increasing number of women from ethnic minorities and underserved groups, there is a clear scope to tailor the MCoC model to these populations, supporting and enhancing their antenatal care experience and promoting a more inclusive, equitable approach to maternity care.

PPI partners acted as research collaborators and provided valuable insights that ensured the findings of this overview were interpreted from the perspectives of women making mode-of-birth decisions. We believe that integrating individuals with lived experiences into the data synthesis process even though requires time and support significantly enhances the research output. This approach grounds the findings in real-world experiences, enriching the analysis with greater cohesion, depth, and authenticity.

### Strengths and limitations

This overview was conducted following current methodological standards by an interdisciplinary team of clinical and methodological experts, as well as independent PPI partners. By concentrating on disadvantaged, marginalised, and ethnic minority women in high-income countries, this overview addresses critical gaps in understanding the unique challenges faced by these populations, thereby highlighting opportunities for equitable care improvements. The clear identification of barriers and facilitators provides actionable insights for policymakers and healthcare professionals to design more inclusive maternity services. It is important to acknowledge that most included reviews were conducted in the UK, potentially limiting the generalisability of findings to other high-income countries with differing healthcare systems and cultural contexts. Moreover, demographic details of participants were often underreported, limiting the ability to fully characterise the study populations. The findings of this overview will inform the development of a decision aid designed to facilitate antenatal discussions within the UK NHS. This tool aims to support informed decision-making for expectant mothers, including those from ethnic minorities and marginalised and underserved groups.

## Conclusions

This overview of systematic reviews highlights the challenges faced by ethnic minorities and underserved, marginalised, and disadvantaged women during antenatal care. To address health inequalities, maternity care must be re-evaluated at multiple levels. Our findings highlight the critical role of empathetic, accessible, and equitable communication for these women and stress the need for high-quality, personalised care encompassing attentive listening, psychological support, clear and unbiased information, and respect for individual choices. Building compassionate relationships, honouring cultural values, and acknowledging the diversity of women’s experiences should be central to positive maternity care. These insights can guide healthcare professionals and policymakers in enhancing the quality and inclusivity of maternity care and promoting woman-centred approaches.

## Data Availability

All data supporting the findings of this overview of systematic reviews are provided within the article, its tables and figures. Open Science Framework OSF: Experiences of Women from Ethnic Minorities and Underserved, Marginalised and Disadvantaged Groups in Communicating with Health Professionals during Antenatal Care: An Overview of Qualitative Systematic Reviews. DOI: https://doi.org/10.17605/OSF.IO/VE2FR ^34^. This project contains the following extended data: Supplementary Material - Search Strategies Supplementary Material 2_Quality Assesment Table.docx OSF: PRISMA Checklist for Experiences of Women from Ethnic Minorities and Underserved, Marginalised and Disadvantaged Groups in Communicating with Health Professionals during Antenatal Care: An Overview of Qualitative Systematic Reviews. DOI: https://doi.org/10.17605/OSF.IO/VE2FR ^34^. Data are available under the terms of the Creative Commons Attribution 4.0 International license (CC-BY 4.0). The original content, analyses, and conclusions presented in this manuscript were conceived and written by the authors. ChatGPT-4o (OpenAI) was used solely as a language-editing tool to help shorten and refine certain sections of text for clarity and conciseness. No part of the intellectual or scientific content was generated by artificial intelligence. The authors take full responsibility for the accuracy and integrity of the work.
