# Supplementary material for: Experiences of women from ethnic minorities and underserved, marginalised and disadvantaged groups in communicating with health professionals during antenatal care: An overview of qualitative systematic reviews
Source: NIHR Open Res. 2026 Jun 2;6:2. Originally published 2026 Jan 7. [Version 2] doi: 10.3310/nihropenres.14096.2 (PMC12917361; doi:10.3310/nihropenres.14096.2)
Supplement: Peer review history

# Reviewer report: Reviewer response for version 2

**DOI:** 10.3310/nihropenres.15587.r40666

**Published:** 2026-06-22

**Authors:** Frances Conti-Ramsden

**Affiliations:** 1 King’s College London, London, UK

I would recommend *Approved with Reservations*, as the authors have not addressed the search strategy timeline. I have no further comments.

Are the rationale for, and objectives of, the Systematic Review clearly stated?

Yes

Is the statistical analysis and its interpretation appropriate?

Yes

Are sufficient details of the methods and analysis provided to allow replication by others?

Yes

Are the conclusions drawn adequately supported by the results presented in the review?

Yes

Reviewer Expertise:

NA

I confirm that I have read this submission and believe that I have an appropriate level of expertise to confirm that it is of an acceptable scientific standard, however I have significant reservations, as outlined above.

# Reviewer report: Reviewer response for version 2

**DOI:** 10.3310/nihropenres.15587.r40664

**Published:** 2026-06-04

**Authors:** Caroline Mitchell

**Affiliations:** 1 Keele University, Newcastle-under-Lyme, UK

I have reviewed the revised version of the manuscript, and I am happy to approve this updated version.

Are the rationale for, and objectives of, the Systematic Review clearly stated?

Yes

Is the statistical analysis and its interpretation appropriate?

Not applicable

Are sufficient details of the methods and analysis provided to allow replication by others?

Yes

Are the conclusions drawn adequately supported by the results presented in the review?

Yes

Reviewer Expertise:

NA

I confirm that I have read this submission and believe that I have an appropriate level of expertise to confirm that it is of an acceptable scientific standard.

# Reviewer report: Reviewer response for version 1

**DOI:** 10.3310/nihropenres.15329.r39294

**Published:** 2026-02-18

**Authors:** Caroline Mitchell

**Affiliations:** 1 Keele University, Newcastle-under-Lyme, UK

A rigorous and comprehensive systematic overview of published systematic reviews has been undertaken which addresses the research objectives to synthesise qualitative evidence on the experiences of women from ethnic minorities and underserved, marginalised and disadvantaged groups in communicating with health professionals during antenatal care. A qualitative meta synthesis in a review of reviews was undertaken so no statistical analyses were undertaken. However, they did tabulate and present numerical data- numbers of participants overall overlap in % terms in content of the reviews. The multidisciplinary team demonstrator's appropriate expertise PPI partners provided complementary insights.

 The findings are relevant to further research being undertaken by the team and to wider clinical contexts.

Are the rationale for, and objectives of, the Systematic Review clearly stated?

Yes

Is the statistical analysis and its interpretation appropriate?

Not applicable

Are sufficient details of the methods and analysis provided to allow replication by others?

Yes

Are the conclusions drawn adequately supported by the results presented in the review?

Yes

Reviewer Expertise:

Professor of General Practice Research and GP. I have experience in undertaking and supervising  systematic reviews- particularly qualitative meta syntheses and also umbrella review methodology. I am a mixed methods research and in particular I have experience in community based participatory research with and by underserved communities. I have topic specific expertise- maternity disparities

I confirm that I have read this submission and believe that I have an appropriate level of expertise to confirm that it is of an acceptable scientific standard.

# Reviewer report: Reviewer response for version 1

**DOI:** 10.3310/nihropenres.15329.r39194

**Published:** 2026-02-12

**Authors:** Frances Conti-Ramsden

**Affiliations:** 1 King’s College London, London, UK

This is a well written systematic review on the topic of minoritised and disadvantaged women’s experiences of antenatal care. The study was pre-registered and is methodologically sound with good involvement of lived experience perspectives throughout the study. The findings are relevant and important for planning of maternity care provision.

 My primary comment is the search time window; the literature search was conducted until 2022. This is now 4 years ago; there may have been substantial additions to the literature in the interim. My recommendation would be for the search to be extended to 2025 to ensure more recent studies are captured.

 In addition, whilst I appreciate the focus of the review is on women’s experiences of antenatal care, I would like to see more in the discussion on the evidence for how women’s experiences shapes their outcomes; what evidence do we have that the experiences described impacts outcomes such as preterm birth, development of pregnancy complications and thereby healthcare associated costs? How do we justify to funders and policy makers that it makes business as well as ethical sense to invest in this problem?

Are the rationale for, and objectives of, the Systematic Review clearly stated?

Yes

Is the statistical analysis and its interpretation appropriate?

Yes

Are sufficient details of the methods and analysis provided to allow replication by others?

Yes

Are the conclusions drawn adequately supported by the results presented in the review?

Yes

Reviewer Expertise:

Hypertensive disorders of pregnancy, Ethnicity and ancestry, obstetrics and maternal medicine. I am primarily a quantitative researcher so I cannot comment on the qualitative thematic analysis.

I confirm that I have read this submission and believe that I have an appropriate level of expertise to confirm that it is of an acceptable scientific standard, however I have significant reservations, as outlined above.

# Reviewer report: Reviewer response for version 1

**DOI:** 10.3310/nihropenres.15329.r39292

**Published:** 2026-01-29

**Authors:** Samuel Oluwatobi Atiku

**Affiliations:** 1 Aston University Birmingham, Birmingham, UK

**General Assessment**

 This manuscript is one of the most robust and professionally executed qualitative overviews of social disparities in antenatal care that I have encountered. The technical rigour is exemplary. The use of the GROOVE tool to confirm minimal primary study overlap of 1.08 per cent and the sustained integration of Public and Patient Involvement partners from the grant proposal stage set a notably high standard. The Results section is the clear strength of the paper, offering a rich and deeply human evidentiary base while remaining methodologically sound.

 Overall, the manuscript is already of a standard that could reasonably be accepted in its current form. However, its impact and clarity could be further enhanced with focused revisions. In particular, the Introduction should more clearly articulate the unique contribution of an overview of reviews and better anticipate the conceptual depth of the findings. Similarly, adding brief synthesising statements in the Results and Discussion would help ensure the themes are framed as structural insights into how communication operates as a mechanism of power, exclusion and inequity within maternity systems. With this reframing, the paper would more fully match the originality and international relevance of the evidence presented.

**Section-Specific Feedback**

**Introduction**

 The Introduction is clearly written and effectively establishes the importance of communication in antenatal care for women from ethnic minority and underserved, marginalised and disadvantaged groups. It provides appropriate background on maternal health disparities and offers a sound rationale for focusing on communication. However, the originality, scope and analytical ambition of the study are not yet fully conveyed.

 First, the critical evidence gap addressed by this overview is not articulated with sufficient clarity. While the Introduction notes the existence of qualitative evidence on the experiences of underserved women, it does not explain why an overview of qualitative systematic reviews is a necessary next step. Numerous reviews focus on specific populations, including migrant women, women with physical disabilities, survivors of female genital mutilation, and trafficked women. However, the fragmentation of this evidence base is not made explicit. Given the minimal overlap between primary studies in the included reviews, the case for a higher-level synthesis to identify cross-cutting, systemic patterns in communication failures could be made more forcefully. As currently framed, the rationale risks reading as procedural rather than as a clear statement of unique contribution.

 Second, communication is conceptualised primarily in practical and functional terms, as a means of information exchange and care personalisation. The findings, however, demonstrate that communication operates as a relational and structural mechanism through which power, discrimination, surveillance and exclusion are enacted within maternity systems. This deeper conceptual dimension is not sufficiently anticipated in the Introduction, creating a disconnect between the framing and the analytical depth of the results. Strengthening the conceptual framing would better prepare readers for the ethical and clinical issues that emerge later in the paper.

 Third, the Introduction places relatively strong emphasis on the contribution of the study to informing the Plan A birth plan decision aid. While this is an important application, its prominence may inadvertently narrow the perceived relevance of the work. Readers, particularly those outside the UK, may interpret the paper as a local programme report rather than a high-level overview with broader international relevance. The synthesis clearly has wider implications for maternity service organisation, professional education and policy responses to maternal health inequities. These should be more explicitly foregrounded.

 Fourth, the methodological ambition of the study is not fully signalled at the outset. The depth of the PPI partnership and its influence on the research question and interpretation are described later but not highlighted in the Introduction. This is a major strength of the work and is currently underplayed. Earlier acknowledgement of this co-production would strengthen the ethical grounding of the synthesis and signal that the analysis is informed by lived experience as well as academic expertise.

 Finally, while maternal health inequalities are noted, the clinical and ethical stakes of communication failures are not made explicit early on. The links between poor communication, disengagement from services, delayed care-seeking and adverse maternal outcomes are implied rather than stated. This reduces the sense of urgency that is evident in the Results and Discussion.

**Recommendation**

*I recommend revising the Introduction to more clearly articulate the unique contribution of this overview, deepen the conceptual framing of communication, broaden the perceived relevance beyond a single applied output, and foreground both methodological rigour and the high stakes of communication failures. These comments should be read not as a critique of the quality of the Introduction, which is strong, but as an opportunity to elevate the framing so it matches the depth, originality and international relevance of the findings.*

**Methodology**

 The Methodology section is transparent, well structured and aligned with recognised reporting standards, including PRISMA 2020 and PROSPERO registration. The integration of a robust PPI framework, with partners involved from the grant proposal stage through to interpretation, is a significant strength that adds credibility to the synthesis. Some procedural and analytical decisions, however, require further clarification.

 First, the eligibility criteria related to setting and clinical profile appear restrictive. The requirement that reviews include at least one UK-based study creates a geographic focus that may have excluded relevant high-income country evidence. In addition, while the criteria specify clinically uncomplicated pregnancies and births after 37 weeks, it is unclear how this was operationalised at the review level. As systematic reviews often include mixed risk profiles, the authors should clarify how this distinction was maintained or acknowledge the limitations of doing so in an overview of reviews.

 Second, the description of the search strategy lacks immediate detail on key limits. Although the full MEDLINE strategy is available externally, the manuscript should explicitly state any language restrictions and whether grey literature was searched. Including this information in the main text would allow readers to more readily assess search sensitivity and potential publication bias.

 Third, greater transparency is needed regarding the handling of population heterogeneity and the synthesis process. The analysis brings together highly diverse groups, including women with physical disabilities, women with obesity, survivors of trafficking and women affected by FGM. The methodology does not fully explain how group-specific mechanisms of exclusion were preserved when developing cross-cutting themes. Further detail is also needed on how findings from different types of evidence products, such as realist or integrative reviews, were translated into a single analytical framework. Clarification on whether dual-author review involved independent parallel coding or a verification approach would further strengthen the section.

 Fourth, the role of quality appraisal and overlap assessment in shaping the synthesis remains largely descriptive. Reviews identified as low quality are noted, but it is unclear how this influenced the confidence assigned to specific themes. Similarly, although the GROOVE tool identified minimal overall overlap, several review pairs showed high overlap. The authors should explain how duplicated primary findings were managed to avoid over-representation.

**Recommendations**

1. *Clinical profile granularity*
* Please explicitly acknowledge the challenge of maintaining a focus on clinically uncomplicated pregnancies at the overview level. This should be noted as a limitation, given reliance on the inclusion criteria of the 19 reviews and the inability to independently verify clinical profiles across the 381 primary studies.*
2. *Geographic scope and framing*
* Clarify the deliberate UK focus either in the Introduction or Methodology. Explicitly state that this decision was made to ensure relevance for the NHS and the Plan A decision aid, while noting that many themes remain transferable to other high-income settings.*

**Results**

 The Results section is the most significant contribution of this manuscript, characterised by a high degree of methodological rigour and a rich evidentiary base. The use of the GROOVE tool to confirm minimal overall primary study overlap (1.08%) is a technical highlight that validates the breadth of the synthesis and ensures the findings represent a diverse spectrum of qualitative evidence. The thematic organisation is logical and effectively captures the complexity of antenatal care experiences. However, the presentation would benefit from minor analytical framing to ensure the nuanced findings are synthesised into clear structural patterns.

 First, the section identifies critical barriers to maternity care, but the framing occasionally stops at descriptive collation. Theme 1 successfully reports widespread communication failures and negative professional attitudes across 14 of the 19 reviews. However, to move beyond a topical list, each subtheme should conclude with an analytical synthesis.

**Recommendation:** *At the end of the ‘Communication’ subtheme, add a sentence identifying communication not just as a failure of clarity, but as a structural gatekeeper that prevents marginalised women from navigating the system with the same agency as the majority population.*

**Recommendation:** *Within the ‘Attitudes’ subtheme, conclude by noting that the ‘processed’ or ‘functional but not supportive’ care reported by women evidences a systemic technocratic priority, where institutional efficiency is frequently valued over the dignity and psychological safety of the patient.*

 Second, the group specific findings for underserved populations are remarkably detailed but require more nuanced framing of the ‘othering’ process. The results for women with obesity, disabilities, and FGM are particularly strong, documenting how clinical focus shifts from the pregnancy to the mother’s physical ‘condition’.

 Recommendation: *This section would be strengthened by a concluding synthesis sentence identifying the cross cutting pattern of medical ‘othering’, where the pathology of the mother repeatedly diminishes her identity and agency as an expectant parent.*

 Third, the findings regarding trust and security reveal profound structural disconnects. You report that fears of deportation or the intervention of Child Protection Services are primary deterrents to seeking care.

**Recommendation:** *Synthesise these findings by noting that for these specific populations, the healthcare system is often perceived as a site of surveillance rather than a site of support, fundamentally altering the nature of the patient professional relationship.*

 Fourth, Theme 2 (Facilitators) identifies actionable interventions but lacks the analytical depth of the Barriers section. While only eight reviews provided data on facilitators, the findings regarding continuity of care and bicultural staff are vital.

**Recommendation:** *Conclude this theme by explicitly linking these facilitators to the structural barriers identified earlier. For example, note that continuity of care and culturally responsive staff serve as the primary mechanisms for dismantling the systemic mistrust and surveillance fears identified in Theme 1.*

 Fifth, minor methodological outcomes in the Results require more explicit reporting to ensure data integrity. The manuscript identifies two ‘low quality’ reviews and a 10% data cross check process, yet the impact of these factors on the final results is not stated.

**Recommendation: **Briefly state whether findings from low quality reviews were weighted differently and report the level of agreement reached during the data cross check to reinforce the dependability of the thematic synthesis.

**Overall judgement:** *These Results provide a robust and deeply human look into antenatal inequality. Adding focused, synthesising sentences at the end of each subtheme will ensure these nuanced findings are framed as the structural and systemic insights they are, rather than a collection of qualitative reports.*

**Discussion**

 The Discussion section provides a comprehensive interpretation of the evidence, successfully bridging the findings from the 19 systematic reviews to the practical requirements of the Plan A study. It effectively synthesises the core barriers such as language, cultural differences, and institutional mistrust into a call for a re evaluation of maternity care delivery. However, to match the analytical depth suggested for the Results, the Discussion should more explicitly address structural power dynamics and the interdependence of the proposed solutions.

 First, the Discussion correctly identifies communication as the central pillar of antenatal care, but it could further explore communication as an equitable process. The authors highlight that interpretation services are currently insufficient in many UK centres and that policymakers must prioritise tailored communication strategies.

**Recommendation**: *While you advocate for ‘empathetic, accessible, and equitable communication’, the Discussion would be strengthened by explicitly framing communication as a redistribution of power. Ensuring women are ‘attentively listened to’ is not just a clinical courtesy but a structural necessity to counter the ‘paternalistic’ and ‘dehumanising’ experiences reported in the Results.*

 Second, the analysis of healthcare professional attitudes provides a clear mandate for training, though it should emphasise systemic change over individual behaviour. The manuscript notes that women felt treated with less dignity due to their background or appearance and highlights the need for cross cultural training.

**Recommendation: T** *he Discussion should emphasise that these ‘rude’ or ‘insensitive’ comments are symptoms of a wider institutional culture. Training should therefore focus not only on individual cross cultural skills but on dismantling the unconscious biases that lead to the medical ‘othering’ identified in the findings for women with obesity and FGM.*

 Third, the recommendation for the Midwifery Continuity of Carer (MCoC) model is a significant strength that directly addresses the findings. You correctly identify that consistent, supportive care is vital for women with past traumatic experiences and that the MCoC model should be tailored to these specific populations.

**Recommendation:** *Explicitly conclude this section by noting that continuity of care is the most effective structural remedy for the surveillance fears identified in the Results. By building a ‘trusting and safe relationship’, the MCoC model functions as a protective shield against the systemic mistrust that currently causes vulnerable women to avoid care.*

 Fourth, the integration of PPI insights adds unique authenticity to the Discussion. The inclusion of PPI partners in the data synthesis process is noted to have enriched the analysis with ‘depth and authenticity’.

**Recommendation:** *The Discussion would benefit from a sentence explaining how the PPI perspective specifically helped the team interpret the nuance of the findings, perhaps by identifying that what researchers call ‘non compliance’, women experience as ‘self protection’ in the face of perceived institutional threat.*

 Fifth, the acknowledgement of limitations is transparent but could be more critical regarding the UK centric evidence base. You note that most included reviews were conducted in the UK, which informs the development of the UK specific decision aid.

**Recommendation:** *Briefly discuss how the UK NHS context, as a universal healthcare system, might uniquely shape these findings compared with other high income settings with insurance based models, ensuring the equitable care improvements you suggest are contextually grounded.*

Are the rationale for, and objectives of, the Systematic Review clearly stated?

Yes

Is the statistical analysis and its interpretation appropriate?

Yes

Are sufficient details of the methods and analysis provided to allow replication by others?

Partly

Are the conclusions drawn adequately supported by the results presented in the review?

Yes

Reviewer Expertise:

My research evaluates social and behavioural disparities and how they interact with public financial management (PFM), governance systems, and institutional processes to influence access to services. I examine mechanisms such as communication barriers, discrimination, and documentation or administrative requirements, and how budgeting, financing, and resource allocation decisions reinforce unequal treatment and outcomes, particularly for disadvantaged populations and in areas affecting health. I also examine the mechanisms through which policies are designed and implemented and how these processes shape real-world outcomes.

I confirm that I have read this submission and believe that I have an appropriate level of expertise to confirm that it is of an acceptable scientific standard, however I have significant reservations, as outlined above.
